# Signals of selection in the mitogenome provide insights into adaptation mechanisms in heterogeneous habitats in a widely distributed pelagic fish

**DOI:** 10.1038/s41598-020-65905-1

**Published:** 2020-06-03

**Authors:** Wilson Sebastian, Sandhya Sukumaran, P. U. Zacharia, K. R. Muraleedharan, P. K. Dinesh Kumar, A. Gopalakrishnan

**Affiliations:** 1 Marine Biotechnology Division, ICAR-Central Marine Fisheries Research Institute, Ernakulam North P.O., Kochi, 682018 Kerala India; 2 CSIR-National Institute of Oceanography, Regional Centre Kochi, Dr. Salim Ali Road, Post Box No. 1913, Kochi, 682 018 Kerala India

**Keywords:** Ecological genetics, Evolutionary ecology

## Abstract

Oceans are vast, dynamic, and complex ecosystems characterized by fluctuations in environmental parameters like sea surface temperature (SST), salinity, oxygen availability, and productivity. Environmental variability acts as the driver of organismal evolution and speciation as organisms strive to cope with the challenges. We investigated the evolutionary consequences of heterogeneous environmental conditions on the mitogenome of a widely distributed small pelagic fish of Indian ocean, Indian oil sardine, *Sardinella longiceps*. Sardines were collected from different eco-regions of the Indian Ocean and selection patterns analyzed in coding and non-coding regions. Signals of diversifying selection were observed in key functional regions involved in OXPHOS indicating OXPHOS gene regulation as the critical factor to meet enhanced energetic demands. A characteristic control region with 38–40 bp tandem repeat units under strong selective pressure as evidenced by sequence conservation and low free energy values was also observed. These changes were prevalent in fishes from the South Eastern Arabian Sea (SEAS) followed by the Northern Arabian Sea (NAS) and rare in Bay of Bengal (BoB) populations. Fishes belonging to SEAS exhibited accelerated substitution rate mainly due to the selective pressures to survive in a highly variable oceanic environment characterized by seasonal hypoxia, variable SST, and food availability.

## Introduction

Adaptive mechanisms are essential in marine fishes to survive in rapidly changing oceans and consequently understanding signals of adaptation will pave the way for sustainable utilization of marine resources. Ecophysiological adaptations at the cellular level provide resilience to the organism and the role of the mitochondrion and mitochondrial genome in these processes has been regarded as pivotal in recent investigations^1–3^. Mitochondria play important roles in the bioenergetics of tissues by producing 95% of eukaryotic cell energy (ATP) through the process of oxidative phosphorylation (OXPHOS) and the five major protein complexes involved in OXPHOS are crucial in the electron transport system and ATP synthesis^4^. Mitogenomic investigations on Pacific salmon revealed that key adaptations in OXPHOS proteins are important in lineage sorting^5^ whilst positively selected sites in cytochrome b region of a widely distributed European anchovy were correlated with thermal clines^6^. Mutations of genes involved in OXPHOS have been correlated with a wide range of environmental factors like hypoxia^7^, heat stress^2^, cold stress^8^, nutrient availability^9^ and the difference in expression of genes^10^. Such mutations have been indicated as providing adaptation to different thermal regimes in Drosophila^11^ and differential aerobic capacity in Killifish^12^. These investigations emphasize the importance of identifying the loci involved in selection as these loci could be used as markers to study environmental adaptation.

Tropical Indian ocean has been warming for over a century at a rate that is faster than any other region of the tropical oceans which influences the sea surface temperature (SST) patterns globally^13^. Seasonality in the Arabian sea was well profound in the horizontal section plots of SST, DO and Chlorophyll-*a*, while its counterpart Bay of Bengal (BoB) showed minimum variation in the above properties. Seasonal dynamics in the Northern Arabian Sea (NAS) has mainly been driven by both monsoonal forcing through intensive mixing and upwelling processes. During the summer monsoon, generally from June-September, Findlater jet^14^ (strong winds that blow from the southwest forming an intense low-level jet) create open ocean upwelling and downwelling in its left and right side of the path, while intense upwelling can be seen along the west coast^15^ and southeastern Arabian Sea^16^. In winter monsoon, evaporative cooling results in high dense water mass in the NAS that sinks to deeper depths and uplift subsurface waters to surface layers^17^ while the southern Arabian Sea becomes stratified due to the intrusion of the BoB waters^18^. Positive water balance in the BoB creates strong and stable stratified surface layers. Weak monsoonal winds^19^ may not be able to break these layers most of the time except, cyclones, strong cold-core eddies, strong winds, etc. Transitional periods such as inter-monsoon spring and intermonsoon fall are associated with primary and secondary heating periods that warm the surface layers and create stratification in both the seas.

The inhabitants of this ecosystem will be under an intense selection pressure to meet the enhanced energetic demands due to the increased SST as well as the changes in salinity, dissolved oxygen, food availability, and hydrological factors^20^. Mitochondrial genome adaptations may provide resilience to these climatic factors by changes in the efficiency of the OXPHOS complex^21,22^, which could be monitored over time to understand spatial and temporal patterns in the distribution of some sentinel species like Indian oil sardine. Further, conservation and management strategies can be devised to protect or conserve the adapted populations ensuring the sustainability of sardine populations, which constitute a cheap source of protein for the burgeoning human population, especially in developing countries like India^23,24^.

Control region, the non-coding content present in the mtDNA is responsible for the regulation of replication and transcription of mitogenome^25^ with many conserved sequence elements/domains, binding sites for nuclear-encoded factors, replication initiation sites, transcription initiation sites, and termination associated sites^26^. Many control region segments can form stable intra-sequence secondary structures^25,27^ which are identified as recognition sites/binding sites for many regulatory proteins like transcriptional factors^25,27,28^.

The Indian oil sardine *Sardinella longiceps* is distributed across wide environmental clines in the Indian Ocean, mainly in the northeast, southeast, southwest and northwest Indian coast, Gulf of Oman and Gulf of Aden^29^. Temperature followed by salinity and dissolved oxygen availability are the vital factors determining the seasonal fluctuations in distribution and abundance of small pelagic fishes like sardines and anchovies^30,31^. The availability of nitrogen from upwelling and sinking events in addition to external sources, especially runoff from rivers^32,33^, also influences the distribution and abundance. Fluctuations in these parameters induce physiological stress and consequent changes in metabolic rate, survival, and persistence. Small pelagic fishes like Indian oil sardine have high-metabolic requirements with high dependence on aerobic metabolism, making them vulnerable to environmental perturbations. Being ectothermic, their body temperature and metabolic rates are highly dependent on sea temperature. Temperature and salinity clines were reported in the Indian Ocean between the Arabian Sea on the west and the Bay of Bengal on the east^34^. The wide distribution of these sardines across these environmental gradients indicates their excellent adaptive capacity to different ecozones and transitional zones. In the present study, we investigated the signals of adaptation in the mitogenome by sampling fishes from widely spaced ecoregions and analyzing selective constraints. Forty-five complete mitogenomes, along with 350 complete mitochondrial control regions were analyzed from Indian oil sardines collected from the Indian ocean, mainly the eastern Indian Ocean (Bay of Bengal) and western Indian ocean (South Eastern Arabian Sea and Northern Arabian Sea). Subsequently, we investigated signals of positive/purifying selection and its correlation with geographical distribution. We also analyzed the mitochondrial non-coding control region/D loop sequences for functional constraints and their geographical significance if any.

## Results

### Mitogenome sequencing and assembly

The size of the mitogenome ranged from 16598 to 16676 bp depending on the size variation in the control region. No identical sequences were found. The maximum likelihood tree of the whole mitogenome sequence revealed different clades with moderate bootstrap support (Supplementary Fig. A1). Descriptive statistics of nucleotide data sets are given in Table 1. The level of nucleotide diversity was low for the whole mitogenome (ranging from 0.0060 to 0.00132) (Supplementary Table T3) with only 1131 segregating sites whereas haplotype diversity was high with each genome representing a unique haplotype (45) as evident in haplotype network (Supplementary Fig. A2). The significant negative Fu’s *F*_*s*_ and Tajima’s *D* for the whole genome (−8.642 and −2.319) and concatenated protein-coding gene data set (−11.318 and −2.370) (Table 1) indicated an excess of rare nucleotide site variants and rare haplotypes respectively compared to what would be expected under neutrality^35^. The values Fu’s *F*_*S*_, Fu & Li’s F*^36^, and Fu’s & Li’s *D* are presented in Table 1.

**Table 1:** Summary of descriptive genetic diversity statistics of entire mitogenome and concatenated protein-coding genes of *S. longiceps* mitochondrial genome.

	*S*	π	No of haplotype	*H*d	*K*	Number of Synonymous sites	Number of Non- synonymous sites	*K*s	*K*a	*K*a*/K*s	Θ	µ relative	Tajima’s D
Genome	1131	0.00606	45	1 (0.005)	100.628	—	—	—	—	—	0.00611	1.5	−2.31923 (*P* < 0.01)
Gene concatenated	859	0.00682	45	1.00 (0.005)	77,901	748	136	0.023	0.001	0.043	0.00688	1.12	−2.37011 (*P* < 0.01)
ATP6	47	0.00599	32	0.940 (0.029)	4.094	38	13	0.015	0.003	0.2	0.00604	0.9	−2.4994 (*P* < 0.01)
ATP8	4	0.00132	5	0.211 (0.080)	0.22	2	2	0.004	0.001	0.25	0.00132	0.22	−1.76368 (0.1 > *P* > 0.05)
CO1	78	0.0039	43	0.998 (0.005)	6.054	66	13	0.013	0.001	0.077	0.00392	0.64	−2.46843 (*P* < 0.01)
CO2	54	0.00558	24	0.824 (0.059)	3.859	32	23	0.014	0.003	0.214	0.00562	0.92	−2.5591 (*P* < 0.001)
CO3	32	0.00455	25	0.001 (0.032)	3.569	29	3	0.014	0.001	0.071	0.00457	0.75	−2.1433 (*P* < 0.05)
Control region	107	0.01597	43	0.998 (0.005)	15.115	—	—	—	—	—	0.01631	2.66	−1.5376 (*P* < 0.10)
CYTB	92	0.00656	39	0.984 (0.013)	7.489	79	16	0.022	0.001	0.045	0.00662	1.08	−2.357 (0.1 > *P* > 0.05)
ND1	87	0.00995	34	0.987 (0.007)	9.7	84	5	0.037	0.001	0.027	0.01008	1.64	−1.9555 (*P* < 0.05)
ND2	100	0.00913	38	0.993 (0.006)	9.544	100	4	0.033	0.001	0.03	0.00925	1.51	−2.2421 (*P* < 0.01)
ND3	21	0.00512	21	0.841 (0.045)	1.781	18	4	0.018	0.001	0.056	0.00515	0.84	−2.1182 (*P* < 0.05)
ND4	143	0.00849	42	0.997 (0.005)	11.719	117	29	0.029	0.001	0.034	0.00858	1.4	−2.3601 (*P* < 0.01)
ND4L	7	0.00133	8	0.362 (0.092)	0.396	7	0	0.005	0	—	0.00134	0.22	−2.0336 (*P* < 0.05)
ND5	170	0.00887	44	0.999 (0.005)	16.293	152	24	0.031	0.002	0.065	0.00898	1.46	−2.3059 (*P* < 0.01)
ND6	24	0.0061	28	0.904 (0.040)	3.184	24	0	0.022	0	—	0.0061	0.99	−1.7519 (0.1 > *P* > 0.05)
12S rRNA	11	0.00265	14	0.612	1.046	—	—	—	—	—	0.0011	0.18	−1.7332
				−0.085									(0.1 > *P* > 0.05)
16S rRNA	74	0.00288	34	0.967	4.857	—	—	—	—	—	0.00289	0.47	−2.572
				−0.019									(*P* < 0.001)
tRNAs	27	0.00117	20	0.761	1.701	—	—	—	—	—	0.00117	0.19	−2.425
				−0.069									(*P* < 0.01)
Whole mitogenome nucleotide sequence								Gene concatenated (13 protein coding genes)					
Fu’s *Fs*	−8.642 (*P* < 0.0001)							−11.318 (*P* < 0.0001)					
Fu and Li’s D*	−3.59983 (*P* < 0.02)							−3.59983 (*P* < 0.02)					
Fu and Li’s F*	−3.75132 (*P* < 0.02)							−3.75132 (*P* < 0.02)					

S = number of polymorphic sites, π = nucleotide diversity, Hd = haplotype diversity, K = average number of pairwise nucleotide differences, Ks = number of synonymous substitutions per synonymous site, Ka = Number of non-synonymous substitutions per non-synonymous site, Θ = theta from S and µ = mutation rate.

### Evidence for natural selection

The relative mutation rate varied between genes. Relative mutation rates (µ relative) calculated for different gene regions indicated ND4 (1.4) and ND5 (1.46) genes as rapidly evolving with the highest number of non-synonymous mutations (29 and 24 respectively) (Table 1). No non-synonymous substitutions were observed in ND4L and ND6 genes. Control region, ND genes and ATPase evolved faster than other regions. Cytochrome c oxidase COX (Complex IV) evolved slower than ND genes. tRNAs and 12 S rRNAs were the slowest evolving genes (Table 1).

Signals of significant selection were evident in many codons among the 3798 codons analyzed. FUBAR analysis identified purifying selection as pervasive in the data set with 680 of the codons significant (Supplementary Table T6). Signatures of positive selection (10 sites) were less prevalent than purifying selection and they were observed in Complex I (ND1, ND2, ND4, and ND5), Complex III (CYT B), Complex IV (C01, CO2, and CO3) and Complex V (ATP6). MEME analysis showed that there are 26 sites under episodic diversifying selection (*P* < 0.1). TreeSAAP analysis detected many significant amino acid physiochemical property changes in the positively selected regions of *S. longiceps* mitogenome, with conservative amino acid changes dominating over radical changes. Among this, only those sites identified as positively selected at least by two methods were selected for further analysis (Table 2).

**Table 2:** Codons that are under positive selection in the mitogenome protein-coding genes of *S. longiceps*.

Gene	Amino acid position	From Codon To Codon	From Amino acid To Amino acid	MEME^a^	FUBAR^b^	TreeSAAP	Distribution of amino acid replacement across the population
				*p*-value	Posterior Probability	Significant properties (category of amino acid changes)	
ND1	29	ATT-TTT	Ile-Phe	0.0224	—	—	SEAS
ND1	30	GAG-TTG	Glu-Leu	0.0006	—	The average number of surrounding residues (7) Chromatographic index (8) Hydropathy (8) Surrounding hydrophobicity (7)	SEAS
ND2	302	CTT- CAA	Leu-Gln	0.0061	—	Polarity (7)	SEAS, NAS
C01	25	CTG- CGA	Leu-Arg	0.011	—	Isoelectric point (6) Polarity (7)	SEAS
C01	114	GGC- GCC	Gly-Ala	0.034	0.9101	—	SEAS, NAS
C01	262	AAT- GAT	Asn-Asp	0.0435	0.9062	—	SEAS, NAS, BoB
C02	50	CTT- CAA	Leu-Gln	0.0005	—	Polarity (7)	NAS
C02	63	GAA-GGA	Glu-Gly	0.0138	—	Compressibility (7)	BoB
C02	152	GTT- TCT, TCC	Val-Ser	0.0006	—	—	SEAS
ATP6	114	GTA- CTA, CTC, GCA	Val-Leu, Ala	0.0345	—	—	SEAS, BoB
ATP6	185	ATT- CAA	Ile-Gln	0.0413	—	—	SEAS
C0 3	16	TGA- GGA, TTA, CGA	Trp-Gly, Leu, Arg	0.0429	0.9897	—	SEAS, BoB
C0 3	117	CCA- TTA, TCT	Pro-Leu, Ser	0.0374	0.9769	—	SEAS, BoB
ND 4	148	ACC- AAC	Thr-Asn	0.0208	—	—	SEAS
ND5	9	TCT- TGA, TAT	Ser-Trp, Tyr	0.0019	—	—	NAS
ND5	97	GCC- GGG	Ala-Gly	0.0015	—	—	SEAS
ND5	98	CTT- GTT	Leu-Val	0.0469	—	—	SEAS
ND5	225	GCC- ACC	Ala-Thr	0.038	0.9055	—	SEAS, BoB
ND5	226	ACG- ACT	Thr-Asn	0.0016		—	SEAS, NAS
ND5	227	GCC- TGC	Gly-Cys	0.0423	0.9809	Refractive index (7)	SEAS, BoB
ND5	228	AAA- AAT	Lys-Asn	0.035	0.9745	Isoelectric point (6)	SEAS, BoB
ND5	236	CCC- TCC,TTT	Pro-Ser, Phe	0.0061			SEAS, BoB
CYTB	70	TGC- TAC, GTC	Cys-Tyr, Trp	0.504	0.9507	Chromatographic index (8) Helical contact area (7) Molecular volume (6) Partial specific volume (7)	SEAS, NAS, BoB
CYTB	250	CTA- CAA	Leu-Gln	0.0493		—	NAS
CYTB	311	AAG- CAG	Lys-Gln	0.0471	0.9739	—	SEAS, NAS
CYTB	320	CTT- ATT	Leu-Ile	0.0439	0.9176	—	SEAS, NAS, BoB

The analysis is based on three selection tests: MEME, FUBAR, and TreeSAAP method. MEME - Mixed Effect Model of Evolution, FUBAR - Fast Unconstrained Bayesian Approximation, NAS (Northern Arabian Sea), SEAS (South Eastern Arabian Sea) and BoS (Bay of Bengal).

Twelve sites were identified as positively selected in mitochondrial complex I (NADH: ubiquinone oxidoreductase) of *S. longiceps* and all of them were located in transmembrane helices (#29ND1, #30ND1, #302ND2, #148ND4, #9ND5, #97ND5, #98ND5, #225ND5, #226ND5, #227ND5 and #236ND5) except one which is in the intra-helix loop (#228ND5) (Fig. 2). Some of these sites occur in the functional domains of mitochondrial complex I especially on ND2 (#302 Leu-Gln located in C-terminus), ND4 (#148 Thr-Asn in proton-conducting membrane transporter (Proton_antipo_M) and ND5 (#97 Ala-Gly, #98 Leu-Val, #225 Ala-Thr, #226 Thr-Asn, #227 Gly-Cys, #228 Lys-Asn & #236 Pro-Ser clustered in Proton_antipo_M & N-terminal (Proton_antipo_N). Position 228 (ND5) plays an important role in proton translocation (as a proton donor or acceptor) and hydrogen bond formation^37^. This position has recorded a shift in an amino acid residue from Lysine to Asparagine in the present study. Asparagine is more polar than lysine which may be important in hydrogen bond formation.

**Figure 1:**
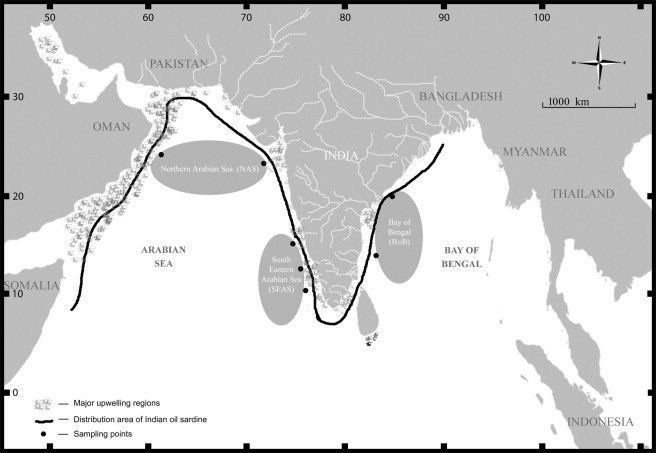
Map showing sampling locations of *S. longiceps* from 3 ecoregions in Indian Ocean. Sample Site: NAS (Northern Arabian Sea), SEAS (South Eastern Arabian Sea) and BoB (Bay of Bengal). The map was drawn using Adobe Photoshop CS6 (https://www.adobe.com/in/products/photoshop.html?promoid=PC1PQQ5T&mv=other).

**Figure 2:**
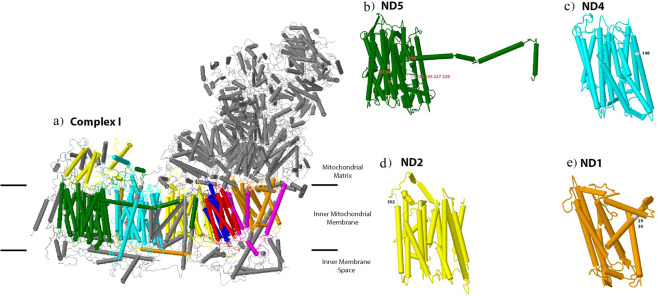
Positive selected sites identified in NADH dehydrogenase (Complex I) of *S. longiceps*. Grey structures represent nuclear-encoded subunits. (**a**) OXPHOS Complex I with mitochondrial-encoded subunits are represented in different colors: ND2 in yellow, ND4L in blue, ND1 in orange, ND3 in magenta, ND4 in cyan, ND5 in green, ND6 in red. Individual core subunits (**b**) ND5, (**c**) ND4, (**d**) ND2 and (**e**) ND1 with amino acid site number on positively selected sites. The protein structures were generated using Geneious R7^88^.

**Figure 3:**
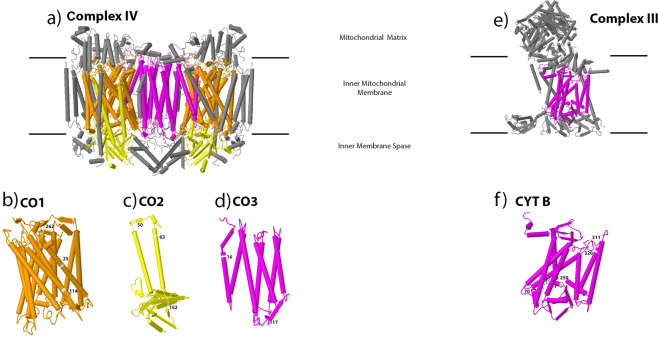
Positive selected sites in Cytochrome C Oxidase (Complex IV) and Cytochrome bc 1 (Complex III) of *S. longiceps*. Grey structures represent nuclear-encoded subunits. (**a**) OXPHOS Complex IV (Homodimer) with mitochondrial-encoded subunits represented in different colors: CO1 in orange, CO2 in yellow, CO3 in magenta. (**e**) OXPHOS Complex III with mitochondrial-encoded subunit represented in magenta color. Individual core subunits (**b**) CO1, (**c**) CO2, (**d**) CO3 and (**f**) CYT B with amino acid site number at positively selected sites. The protein structures were generated using Geneious R7^88^.

Twelve sites were observed to be under positive selection in mitochondrial complex IV (3 sites in CO1; 3 sites in CO2 and 2 sites in CO3). The sites in CO1 (#25Leu-Arg, #114 Gly-Ala and #262Asn-asp) are located in the transmembrane helix and two of these sites (#25 & #114) align with amino acid residues that have been reported to participate in polypeptide binding at Subunit I/VIIc interface & Subunit I/VIIa interface respectively^38–40^. Among three sites observed under positive selection in the CO2 gene, amino acid position 50 (Leu-Gln) lies in the intra-helix loop, position 63 (Glu-Gly) in the transmembrane helix and 152 (Val-ser) in the Beta strand. Among the two sites identified in CO3, position 16 (Trp-Gly) is aligned with the transmembrane helix and position 117 (Pro-Leu, Ser) with the intra-helix loop (Fig. 3).

Four sites were under positive selection in mitochondrial complex III (#70 Cys-Trp, #250 Leu-Gln, #311 Lys-Gln, and #320 Leu-Ile in CYTB gene). Among these sites, one (#311) aligned with an amino acid residue reported to participate in polypeptide binding in the inter-chain domain interface and it is located in the transmembrane helix (Fig. 3). Two sites were under positive selection in mitochondrial complex V (#114 Val-Cys, ala #185 Ile-Gln in ATP6) with site #114 located in the transmembrane helix-4 and the other site #185 in the intra-helix loop connecting helix-5 and 6 (Supplementary Fig. A8).

**Figure 4:**
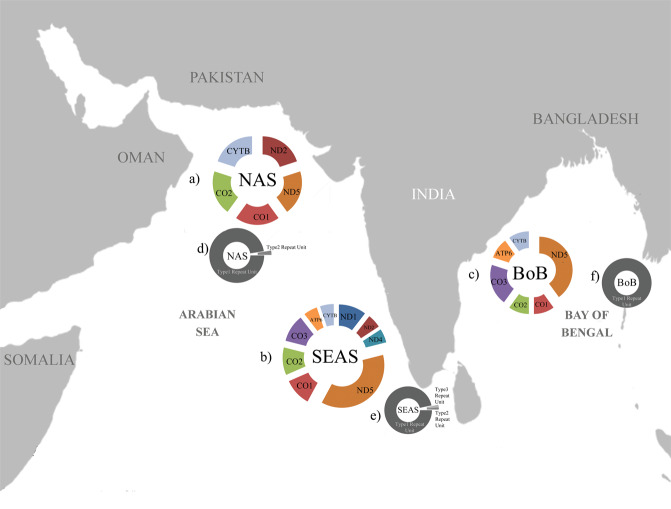
Graphical representation of the geographical distribution of positively selected sites and Control region repeat unit types in the mitogenome of *S. longiceps* in the 3 eco-regions of the Indian Ocean, (**a**) Frequency of positively selected sites in NAS, (**b**) Frequency of positively selected sites in SEAS, (**c**) Frequency of positively selected sites in BoB, (**d**) Frequency of haplotype with Type 1 repeat unit in NAS, (**e**) Frequency of haplotype with Type 2 repeat unit in SEAS and (**f**) Frequency of haplotype with repeat unit Type 3 in BoB. The map was drawn using Adobe Photoshop CS6 (https://www.adobe.com/in/products/photoshop.html?promoid=PC1PQQ5T&mv=other).

Individuals with positively selected sites were prevalent in the South Eastern Arabian Sea (SEAS) samples. Around 26 sites are identified as positively selected in all populations. Among those sites, 81% were observed in individuals from SEAS, 39% from NAS, and 35% from BoB with some of the sites shared among regions. Seven positively selected sites (Two each in ND1 and ND5 genes and one each in ND4, CO1 and CO2 respectively) were specific to SEAS and two sites (one each in CO2 and CYTB) to NAS populations (Table 2; Fig. 4) indicating the presence of locally adapted variants.

### Types of repeat units in the mtDNA control region

The length of the control region of *S. longiceps* ranged from 900−980 bp (GenBank Accession No: KJ466087–KJ466091; KJ472113–KJ472120; KJ888156–KJ888390; KP000859–KP000897), due to variation in the number of tandem repeats and poly-A in different haplotypes (Supplementary Fig. A4). The control region contained different conserved sequence regions like Termination Associated Sequence (TAS) at the 3′ end and Conserved Sequence Box (CSB D, CSB1, CSB2, and CSB3)^41^. (Supplementary Fig. A3). Two hundred and fifty-nine haplotypes with 3 types of repeats were found among the 304 control regions analyzed. The tandem repeat was found in between TAS and poly-A (length between 38–40 bp), the repeat units were repeated once (in the majority of haplotypes), twice and three times (in few haplotypes). Type 1 with one repeat unit (38 bp) was the most abundant (present in all the ecoregions). Type 2 (38 bp) with two repeat units and type 3 (40 bp) with three repeat units were found only in a few individuals (from SEAS and NAS). There are sub-types for Type 3 with some variation in the repeat unit (Type 3a, 3b, and 3c).

### Patterns of the predicted secondary structure of the control region

**Figure 5:**
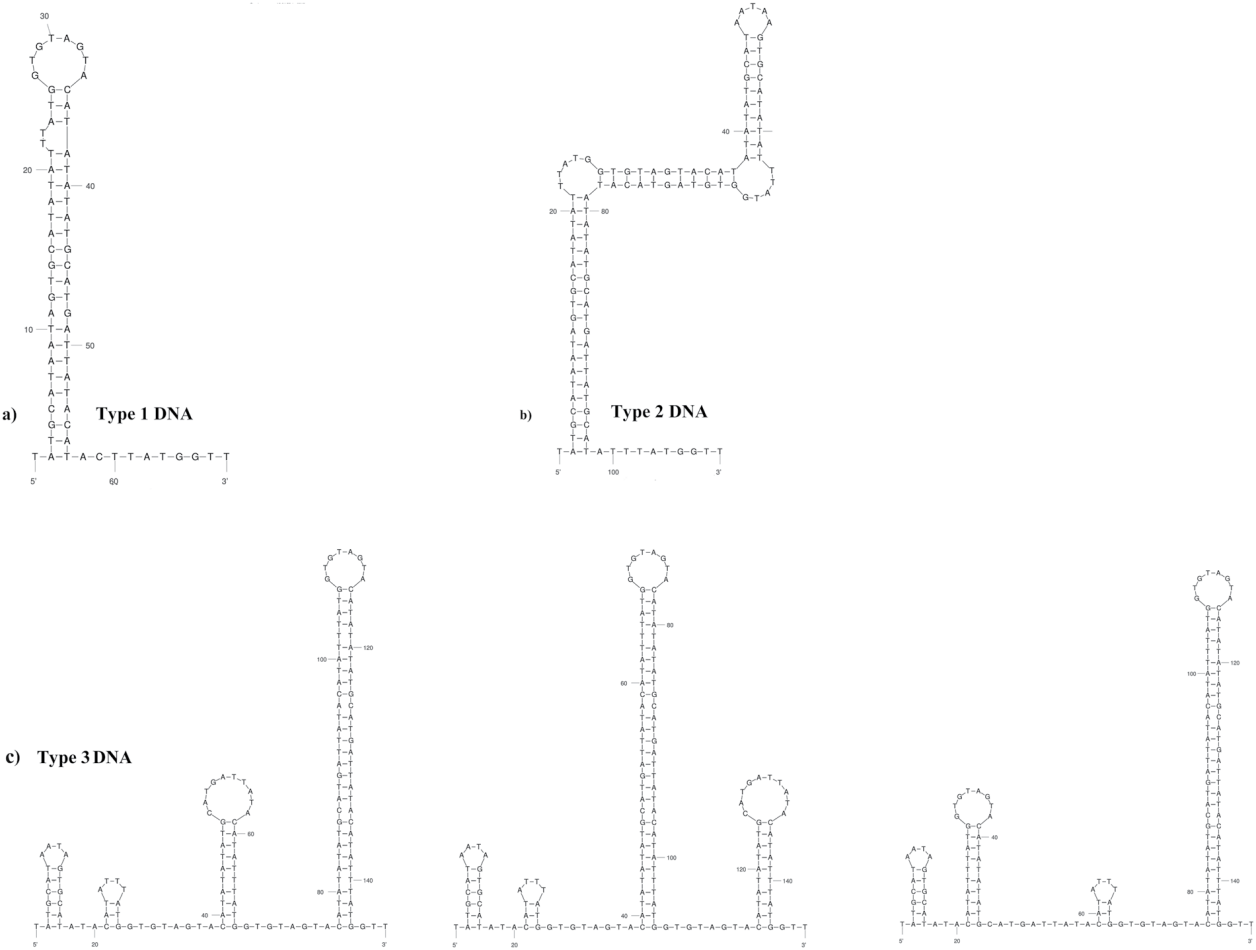
Graphical representation of some predicted secondary structures of repeat unit Type 1, 2 and 3 of mtDNA control region. a) Predicted secondary structure of repeat unit Type 1, b) predicted secondary structure of repeat unit Type2, c) predicted secondary structures of repeat unit Type 3. The secondary structures were generated using ‘mfold’ web server^97,98^.

Several secondary structures (size of 10 bp or more) were identified in the control region L-strand (Supplementary Fig. A5). The conserved sequences like TAS and CSBs were always associated with a secondary structure. All secondary structures predicted for the mtDNA L-strand were also observed for the L-strand mRNA transcript with some minor changes. Palindromic sequences were observed in the region with length variation and repeat units. Few large and short stem-loop structures with low free energy ∆G (−0.101 to −0.384 kcal/mol) were observed in the repeat region (Fig. 5; Supplementary Table T7, Fig. A10). Multiple stem-loop structures have been observed in haplotypes with Type 3 repeat unit sequences whilst, no complex structure observed in Type 1 and Type 2 haplotypes. The stem was formed by the 3′ end of the repeat unit and 5′ upstream sequence. In Type 3 repeat unit, several stem-loop structures with internal bulges were observed. The L strand mRNA transcript of the repeat region is also forming similar structures with greater negative folding energies (∆G) (Fig. 5; Supplementary Table T7, Fig. A10).

The robustness of the predicted secondary structure of the control region was assessed by comparing its folding potential (Free energy, ∆G = kcal/mol) with that of tRNA, which is confirmed to form a functional secondary structure. The relative free energy (∆G/Length) of tRNA ranged from −0.42 to −0.08 and that of predicted repeat unit structures of control region from −0.384 to −0.101 (Supplementary Table T7). Type 1 and Type 2 repeat units have recorded lower free energy than Type 3 repeat units (Supplementary Table T7), indicating the higher folding potential of haplotypes with Type 1 and Type 2 over Type 3 repeat units. Type 1 haplotypes with one repeat unit were the most abundant in all geographic locations of the Indian Ocean. Haplotypes with two and three repeat units were less abundant and restricted to the Western Indian Ocean (both eco-regions) (Fig. 4).

The Tajima’s D value was significant (zero/negative) for tRNA and coding regions, which indicated their functional constraints (Table 1). The mitochondrial control regions involved in secondary structure formation also recorded negative and highly significant values (Supplementary Fig. A3). The repeat unit position alone indicated a value of −2.14526 (P < 0.01) (Supplementary Fig. A3). These results suggest that some regions in the control region (TAS, CSD, and repeat unit) are also under purifying selection force similar to the coding region. The number of substitutions/rates of evolution in paired sites was comparatively lower than the unpaired sites.

### Environmental data

**Figure 6:**
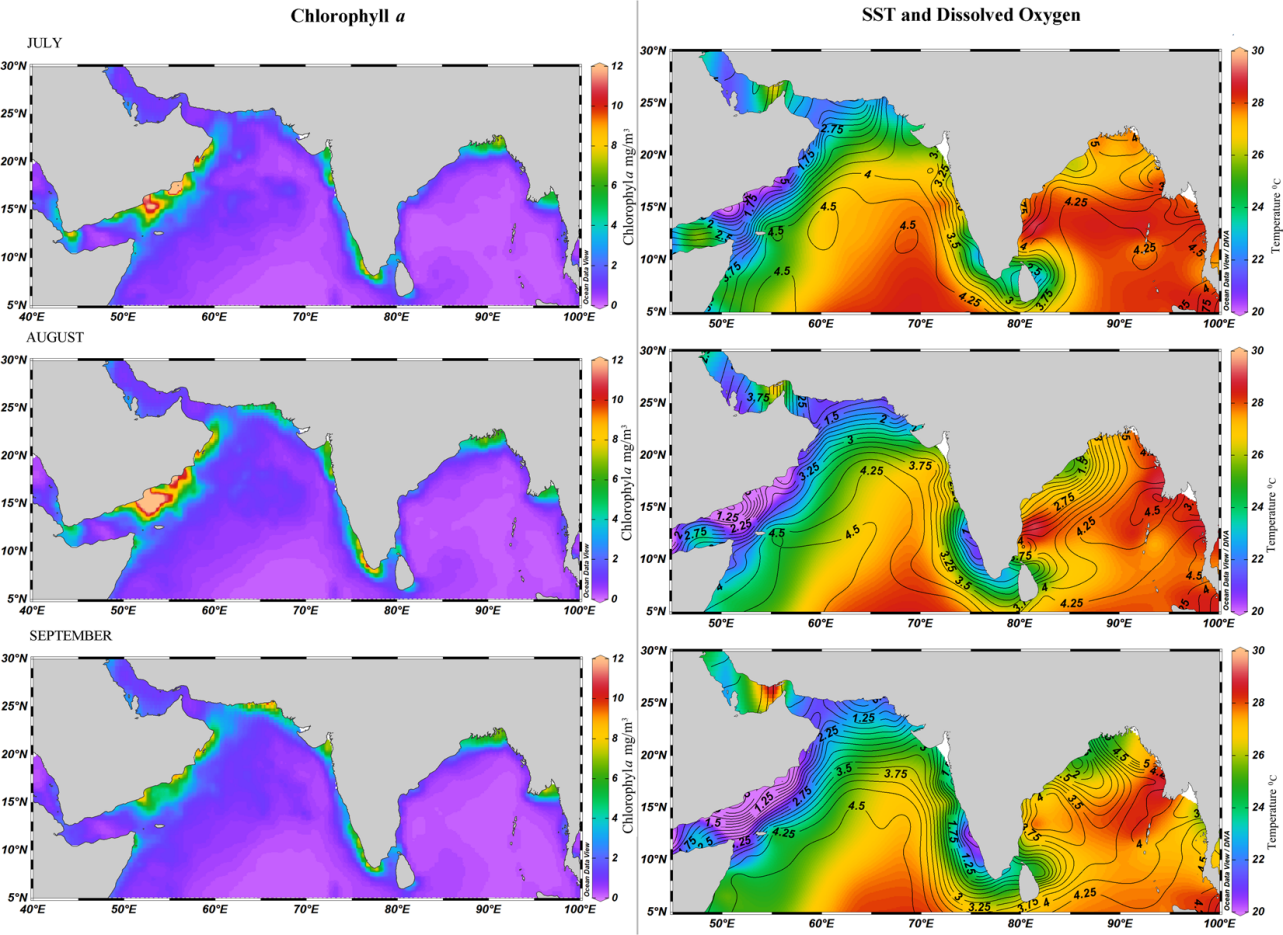
Monthly Chlorophyll *a* (mg/m^3^), Sea Surface Temperature- SST (°C) and Dissolved Oxygen (µmol/kg) for the Bay of Bengal and Arabian Ocean during July to September. Chlorophyll *a* and Sea Surface Temperature gradients are represented as coloured shades. Dissolved Oxygen is represented as contour lines. The images were generated in ODV 5.1.7 (https://odv.awi.de/).

Wide variations in temperature, dissolved oxygen, salinity, and chlorophyll*-a* were observed between NAS, SEAS, and BoB. NAS encompasses the Persian Gulf, Gulf of Oman, Red Sea and the northeast Arabian Sea where a unimodal pattern of sea surface temperature (SST) is observed with the highest temperature (24–27 °C) during the northeast monsoon season (October-March) and lowest temperature (20–22 °C) during the southwest monsoon season (June–September). The NAS also is characterized by a very high chlorophyll*-a* concentration (4–10 mg/m^3^) during May-June, lasting up to October (during the southwest monsoon season). The average sea surface salinity (SSS) is also higher along with NAS throughout the year (36–38ppt)^34^ (Fig. 6, Supplementary Table T5, Fig. A12). SEAS exhibits a typical bimodal pattern of SST with the warm (29–30 °C) spring intermonsoon (April–May) and the fall intermonsoon (October–November) and the cool (26–28 °C) southwest monsoon (June–September) and the northeast monsoon seasons (December-March) (Fig. 6, Supplementary Table T5, Fig. A12). High chlorophyll*-a* concentration (Fig. 6, Fig. A12) observed at SEAS (Malabar upwelling zone) is due to the intense coastal upwelling from May to September, and it peaks during July and August (5–10 mg/m^3^). By October, it recedes to a low (1–2 mg/m^3^) chlorophyll*-a* concentration and maintains up to May. SEAS is also characterized by a very low dissolved oxygen (1–2 mg/L) during the southwest monsoon season while it is 2–4 mg/L in NAS and 3–5 mg/L in BoB. Coastal upwelling along the Somalia coast and SEAS brings not only subsurface cool, nutrient-rich waters but also less oxygenated waters to surface layer, while coastal upwelling regions in the BoB are well oxygenated (Fig. 6, Fig. A12). During this season, high chlorophyll*-a* concentration along the coast is well corroborated with low temperature and low dissolved oxygen. On the contrary, BoB is characterized by stable dissolved oxygen (3–5 mg/L), reduced salinity (28–33ppt), reduced temperature (28–30 °C), and low chlorophyll-*a* (0–3 mg/m^3^) environment than SEAS and NAS, throughout the year. Variations in frequencies of the positively selected amino acid substitutions of the dataset between SEAS, NAS and BoB were positively and highly correlated to fluctuations in annual Sea surface Temperature (SST) (represented as standard deviation) (parameter estimate = 2.04, SE = 0.04, P = 0.01), fluctuations in annual chlorophyll*-a* (represented as standard deviation) (estimate = 1.24, SE = 0.23, P = 0.03) and negatively to Fall (October, November, December) - Dissolved Oxygen (DO) (estimate = −4.68, SE = 0.27, P = 0.02). Moderate correlation was obtained for the amino acid substitutions under selection with fluctuations in annual DO (represented as standard deviation) (estimate = 0.99, SE = 0.4, P = 0.057), Winter -SST (estimate = 0.47, SE = 0.031, P = 0.057), Spring-DO and Summer -Chlorophyll-*a* (estimate = 2.13, SE = 0.13, P = 0.057).

## Discussion

The highly variable environment of the SEAS induced maximum selective pressures in the mitogenome of Indian oil sardine indicating the importance of variable environmental factors on mitogenomic evolution and subsequent adaptation. The positive and diversifying selection was detected for fixed amino acid replacements in key regions involved in oxidative phosphorylation complexes, Complex I: NADH dehydrogenase (ND1, ND2, ND4, and ND5), Complex III: Cytochrome b (CYT B), Complex IV: Cytochrome C Oxidase (CO1, CO2, and CO3) and Complex V: ATP Synthase (ATP6 gene). Seven positively selected sites were specific to SEAS and 2 sites to NAS eco-region. *S. longiceps* has a characteristic control region with 38–40 bp tandem repeat units (palindromic sequences within it) and they are under intense selective pressure similar to the coding region. We predicted stable intra-strand secondary structures with high folding potential/low free energy (−0.101 to −0.384 kcal/mol) in the repeat unit. Haplotypes with one repeat unit are the most abundant haplotypes in the samples probably due to the lower free energy and consequent enhanced stability (high folding potential). Haplotypes with two and three repeat units are less abundant and they are restricted to the SEAS. The observed genetic diversity and positive selection in the mtDNA of *S. longiceps* may be driven by the pressures of the heterogeneous environment. High selective pressures were evident in both coding and non-coding regions in samples collected from SEAS followed by NAS. Variation in frequencies of the positively selected sites between SEAS, NAS and BoB correlated significantly with fluctuations in environmental parameters mainly temperature, dissolved oxygen and chlorophyll-*a*. The SEAS is considered as a region with a complex interplay between many oceanographic processes that vary spatially and temporally^42^.

The NADH dehydrogenase complex is the first and largest multimeric enzyme of the five complexes constituting the oxidative phosphorylation pathway^43^. It provides electrons for the reduction of quinine to quinol which is available from oxidation of the NADH and translocates four protons (H^+^) across the inner membrane. The subunits ND2, ND4, and ND5 directly act as proton pumps for H^+^ ions and the changes in amino acids may have some adaptive value. Among the nine positively selected sites in *S. longiceps*, all of them were located in transmembrane helices especially in proton-conducting membrane transporter. Extensive non-synonymous mutations have been reported in ND genes in many fishes^5,44–47^. The high rate of mutations observed in complex I may be associated with the position of the ND genes in the mitogenome. They are found immediately upstream from the origin of L-strand replication (OriL) and downstream from the origin of H-strand (OriH) replication. During replication, these genes stay single-stranded for more time compared to other genes, thus they are prone to a high rate of mutation^48^.

Cytochrome b is a part of respiratory protein complex III, which is the middle component of the mitochondrial respiratory chain, coupling the transfer of electrons from ubihydroquinone to cytochrome c with the generation of an electrochemical gradient across the mitochondrial membrane. Substitution at amino acid position 311 reported to participate in polypeptide binding in inter-chain domain interface may influence the structure and function of cytochrome b. The amino acid replacements can result in regional changes to hydrophobicity and structure within the protein and can alter the coupling efficiency of complex III. In humans, mutations characterized by enhanced binding of water at Qi site have been linked to increased longevity^49^ whereas, in yeast, mutation at Qo binding site have been linked to reduced catalytic efficiency and increased oxygen radical production^50^. Thus, the substitution at polypeptide binding site of Cytochrome b in the Indian oil sardine may have some functional relevance which needs further investigation.

Cytochrome c oxidase (complex IV) catalyzes the final step in the mitochondrial electron transfer chain and is considered as one of the major regulation sites for OXPHOS^40^. It receives an electron from each of the four cytochrome c molecules which transfers electrons between complex III and IV and transfers them to one oxygen molecule. During this process, it converts one molecular oxygen to two molecules of water by using four protons from the inner aqueous phase to make water and also, translocates four protons across the membrane. The conserved nature of most of the key amino acid residues reported to participate in the electron transfer pathway, putative water exit pathway, ion/chemical binding and putative proton exit pathway in complex IV indicates that these regions are constrained functionally. Mutations observed outside the key functional residues could be related to relaxed purifying selection^49^.

Polymorphisms in regions reported to participate in polypeptide binding at mitochondrial and the nuclear-encoded subunits interface (Subunit I/VIIc interface & Subunit I/VIIa interface; complex IV)^38–40^ may change the structure and efficiency of the OXPHOS complex possibly playing a role in adaptation. Thus, the co-evolution between mitochondrial and nuclear-encoded subunits due to genome-genome interactions can affect the OXPHOS function and regulation in *S. longiceps*. Such co-evolution has been reported in cytochrome c oxidase (complex IV) of primates^51^ and NADH dehydrogenase complex of humans^52^. Positively selected sites that appear to interact with other COX subunits (Complex IV) were also reported from high-performance fish like *Scombroidei*^53^.

ATP synthase (complex V) is composed of a soluble catalytic F1 region and a membrane-inserted F_O_ region. The observed amino acid replacement in the ATP 6 gene (corresponding to a subunit), located in the transmembrane helix-4 (#114) and coil connecting helix-5 and 6 (#185) may have a role in the arrangement of a-helices, which is functionally important and likely involved in proton translocation.

In a population with low effective population size (*N*_e_), fixation of slightly deleterious mutations by drift may leave similar signals as positive selection^54^. But in a pelagic species like *S. longiceps* with large *N*_e_ (*N*_e_ mtDNA 1.1 × 10^6^ to 1.31 × 10^9^)^55^, the effect of genetic drift will be minimal in mtDNA evolution^54^. A previous investigation using two mitochondrial DNA markers (partial gene regions) indicated a lack of significant genetic differentiation in Indian oil sardine populations along the Indian coast^55^ which may be due to the low resolving power of the markers^56^. On the contrary, microsatellite markers indicated significant genetic differentiation between populations of NAS and other regions^57^. Similarly, the evidence of positive selection in regions participating at mitochondrial and the nuclear-encoded subunits interface interactions (CO1, Subunit I/VIIc interface & Subunit I/VIIa interface in complex IV) from SEAS and NAS signals possible reproductive isolation, sympatric speciation and diversification in sardines. Polymorphisms in regions involved in mito-nuclear interactions may disrupt mito-nuclear interactions resulting in reproductive isolation and speciation^58^. Further investigations using genome-wide markers like SNPs may provide more clarity to these findings.

The distribution of mitochondrial control region haplotypes also corroborated the conclusions made out of functional region analysis as all the three types of control region were present in SEAS. The haplotypes with repeat units having the highest folding potential and low free energy are abundant in all oceanic regions. The distribution of other haplotypes with more than one repeat unit is restricted to the SEAS. The folding potential, the number of substitutions/rates of evolution in paired sites, and Tajima’s D statistics analysis showed that control regions are under strong functional constraints. Haplotypes having one repeat unit (Type 1; Supplementary Fig. A10-A), with lower free energy ∆G (high folding potential), were the most abundant. The haplotypes with two (Type 2; Supplementary Fig. A10-B) and three repeat units (Type 3; Supplementary Fig. A10-B) have greater folding energies and they are less abundant and restricted to the SEAS. Different models such as slipped-strand mispairing^59^, intermolecular recombination, transposition^60^, and misalignment during replication have been suggested as the mechanism behind observed polymorphisms^25^. In mammals, these deletions are closely linked to mitochondrial diseases and proven to be associated with site-specific breakage hotspots^59^.

The TAS, poly-A, and secondary structure-forming repeat units were conserved within species. The loop forming regions are protected from mutations that are more likely to occur during replication as it forms a single-stranded structure (D-loop). This strongly indicates the purifying selection pressure on these regions to maintain the intra-strand loop structure and the significant negative Tajima’s D strongly corroborates this hypothesis. The position of the loop forming region between TAS and poly-A indicates its possible role in replication initiation and termination of elongation in proposed models of mitochondrial replication^61^. The occurrence of the secondary structure near the hot spot of polyadenylation sites (Poly A)^62^ strengthens its possible role in transcription termination. The secondary structure may act as a punctuation point for correct mRNA processing^63,64^. The link between mitochondrial structural variants/haplogroups and mtDNA copy number variation (by influencing the replication machinery) has been reported in humans contributing to the adaptation of the human populations to different climatic zones^22,65,66^.

Theoretical and empirical evidence has suggested the role of many environmental parameters for species persistence, adaptation, and phenotypic and genotypic diversification^67,68^. The temperature has been proposed as one of the main factors driving evolutionary diversification due to enhanced mutation rates in mitochondrial as well as nuclear genome in many taxa, which also is the reason for species diversity in tropics as compared to temperate waters^69,70^. It has been demonstrated that fluctuations in the environmental factors promote phenotypic and evolutionary diversification in organisms^71–73^. The Arabian Sea Large Marine Ecosystem is considered as one of the major upwelling systems in the world causing variations in temperature, dissolved oxygen, salinity, and chlorophyll*-a*. SEAS exhibit wide fluctuations in temperature values annually as compared to NAS and BoB. Mitochondria play essential roles in aerobic metabolism which is a temperature-sensitive process and consequently, mitochondria are considered as possible sites of processes influencing the thermal limits of organisms. Thus, thermal acclimation alters mitochondrial properties to maintain aerobic scope^74^. Thermal acclimation in ectotherms may happen by maintaining the stability of OXPHOS proteins for which a few amino acid substitutions may be necessary^75,76^. SEAS is also characterized by variations in chlorophyll*-a* values and oxygen minimum zones (hypoxia). Starvation (reduced chlorophyll-*a* in this case) can drive mtDNA evolution by acting as a force to generate energy more efficiently by improving the efficiency of the coupling of energy production in the OXPHOS pathway^77–79^. Hence, productivity variations of the sardine habitat may lead to the evolution of genotypes with more efficient OXPHOS pathways. Similarly, the occurrence of oxygen minimum zones and consequent hypoxia in SEAS (during southwest monsoon) also demand efficient coupling of energy production^80^. Thus, the abundance of selective signatures/higher rate of selected genotypes in SEAS may be a response to the uncertain environmental conditions (hypoxia, temperature, and productivity) which warrant ecotypes of high metabolic efficiency for survival and reproduction, compared to the stable environment of NAS and highly stable environment of BoB.

There are many reports regarding the correlation between genetic diversity of OXPHOS genes, and environmental pressures such as hypoxia^9^, heat stress^81^, cold stress^8^, and nutrient availability^9^. Adaptive evolution in mitogenome in response to temperature and salinity has been reported in, Atlantic salmon, Pacific salmon, Atlantic cod and Killer whale populations^5,45,82,83^. Thus, the prevalence of diversifying selection in the SEAS indicates the action of evolutionary forces in the mitochondrial OXPHOS complex associated with metabolic adaptation to the dynamic and highly productive environment. Two positively selected sites in ND1 (#29,#30) and ND5 (#97, #98) genes and one site each in ND4 (#148), CO1 (#25), CO2 (#152) and ATP6 (#185) respectively were specific to SEAS and one site each in CO2 (#50) and CYTB (#250) specific to NAS populations. These functional genes and regulatory elements have the potential to act as markers for inferring population genetic structure, plastic responses, adaptation, and functional gene evolution in marine fishes and thus could be valuable for the management and conservation of this important resource. Further studies could be carried out in Indian oil sardine to identify genome level adaptations that will provide holistic information concerning their adaptive capacity. The accelerated warming events in the Indian Ocean necessitate species with adaptive potential and enhanced fitness to increased SST and associated changes in hydrography. The present study assumes great relevance from this point of view as Indian oil sardines with adaptive signals can be further monitored for their spatial and temporal distribution to derive clues regarding climatic impacts in the Indian ocean. Further, information from genomic and mitogenomic investigations can be correlated with fitness consequences at each eco-region by common-garden experiments^83,84,85^. The locally adapted populations will exhibit higher fitness in each eco-region. These investigations are vital to conserve and understand the dynamics of small pelagic resources in space and time as they form the mainstay of food security of many coastal states of developing nations.

## Materials and Methods

### Sample collection, DNA extraction, mitogenome sequencing, and assembly

A total of 350 individuals of the Indian oil sardines were collected during 2015–2017, from the three eco-regions of the Indian Ocean, NAS (117nos), SEAS (117nos), and BoB (116nos) (Fig. 1; Supplementary Table T2). Muscle tissues stored in 95% ethanol were used for genomic DNA extraction using DNEASY blood and tissue kit (Qiagen). All the fishes sampled in this study were handled in strict accordance with the guidelines for the care and use of fish in research by De Tolla *et al*.^86^ and the protocols were approved by the ethical committee of the ICAR- Central Marine Fisheries Research Institute, Kochi.

Complete mitochondrial genomes of 45 individuals (15 each from the 3 eco-regions) were amplified and sequenced using 16 novel primer pairs (Supplementary Table T1) and the mitogenome of *Sardinella longiceps* was used as a template (GenBank Accession No: KR000002.1)^41^. Sequences were manually checked, aligned, and assembled in MEGA6^87^ and Geneious R7^88^ against the *S. longiceps* mitogenome^41^. Annotated mitogenome sequences have been submitted to NCBI, GenBank (Accession numbers MG251937–MG251981). In addition to mitogenome sequences, we also generated control region sequences of an additional 305 individuals to understand structural variations and selective constraints.

Four sets of nucleotide sequence data (1. whole mitogenome nucleotide sequence - 16598 to 16676 bp. 2. concatenated dataset of 13 protein-coding genes - 11418 bp, 3. 22 tRNAs and 4. control region - 1032 to 1108 bp) along with amino acid sequence data (amino acid sequences of 13 protein-coding genes) were prepared and analyzed using MEGA6 and Geneious R7. Descriptive statistics, the number of polymorphic sites (*S*), nucleotide diversity (π)^89^, haplotype diversity (*H*_d_)^89^, the average number of pairwise nucleotide differences (*K*)^90^, Fu’s *F*_*S*_, Fu & Li’s F*^36^ and the total number of synonymous and non-synonymous mutations were generated for nucleotide data sets. The number of non-synonymous substitutions per non-synonymous site (K_a_), number of synonymous substitutions per synonymous site (K_s_), K_a_/K_s_^89^, and theta (θ) were calculated^91^ . The θ-values were used to calculate the relative mutation rate of individual genes relative to the whole mitogenome using the equation µ_gene_ = ((µ_mitogenome_* θ_mitogenome_)/θ_gene_)^46^. All these analyses were performed using DnaSP^92^ and MEGA6. The maximum likelihood tree was generated using MEGA6 for nucleotide and amino acid data sets with 1000 bootstrap replicate and GTR substitution model (selected using the J Model Test^93^). *Sardinella maderensis* sequence was used as an outgroup to root both the trees.

### Selection analyses

The concatenated dataset of 13 protein-coding genes and the Maximum likelihood tree generated from it was used to conduct whole mitochondrial genome scans to detect signals of natural selection. We analyzed the data with the approximate hierarchical Bayesian method (FUBAR- Fast Unconstrained Bayesian Approximation) and mixed effect method (MEME- Mixed Effect Model of Evolution) available in DATA MONKEY^94^. The MEME analyzes the distribution of synonymous and non-synonymous substitution rates from site to site and branch to branch at a site. But FUBAR is considered as more dependable when the strength of selection varies across sites because it uses settings that are less sensitive to model specifications. HKY 85 nucleotide substitution model was used for analysis. For each method we selected a threshold *P*-value; *P* < 0.05 for MEME and posterior probability >0.9 for FUBAR. We used TreeSAAP^95^ to understand changes in physicochemical properties of amino acids caused by replacements, as it compares the amino acid changes inferred from a given tree with a model having 31 predicted physicochemical amino acid property changes, under an assumption of neutrality. The Z test was used to analyze the changes in the amino acid properties, which is categorized into eight magnitude groups^96^. The positive and negative Z-scores indicate positive and negative selection respectively. In this analysis, we considered only 6, 7, and 8^th^ category amino acid changes with strong statistical support (*P* < 0.001).

All amino acid data sets were aligned in MEGA6. The 3D homology model of protein subunits with positively selected sites observed was constructed with the SWISS-MODEL server^97^ using the vertebrate protein model of Bovine corresponding to subunit (available in Protein Data Base, https://www.rcsb.org/). Finally, we located positively selected sites identified in the three-dimensional structure of protein subunit and compared it with the functionally important amino acid residues. The number of positively selected sites was compared between eco-regions to correlate it with habitat characteristics and identify any signals of local adaptation.

### Control region, tRNA sequence analysis, and secondary structure prediction

Repeatability of Sanger sequencing of the control region of three major types was assessed by sequencing the same individual twice so that these sequences could be treated as control samples. Based on the number of repeat units, we elucidated the types of control region sequences present in the dataset. Prediction of the secondary structure of control region sequences was carried out by the free energy minimization method following nearest neighbor thermodynamic rules (with 15 window length and 25 step size) using ‘mfold’ webserver^98^. RNA dataset of control region types and 22 tRNA was generated using MEGA6. The structure and free energy for the RNA datasets were calculated using RNA mfold in ‘mfold’ web server.

We calculated Tajima’s D and the relative mutation rate in control region data to understand the deviation from neutrality/functional constraints on the control region. Sequences spanning the secondary structures were tested for the extent of conservation by comparing relative mutation rates and polymorphisms. Inter-specific comparisons of control region sequences were carried out with those of Clupeoids (using mitogenome sequences deposited in NCBI, GenBank) to deduce identity, sequence conservation, and polymorphisms. All these analyses were performed using DnaSP and MEGA6.

### Environmental data

Monthly climatology data of Sea surface Temperature SST (°C) SSS (ppt) and Dissolved Oxygen DO (µmol/kg) was taken from World Ocean Data 2018 available at https://www.nodc.noaa.gov/OC5/woa18/woa18data.html. While monthly average Chlorophyll*-a* (mg/m^3^) data spanning from the year 2002 to 2015 was downloaded from MODIS site (https://modis.gsfc.nasa.gov/data/dataprod/chlor_a.php) and subjected to objective analysis before generating monthly climatology. The data was analyzed by using Ferret and visualized in Ocean Data View (ODV 5.1.7). Seasonal climatology data (Winter - (January, February, March), Spring - (April, May, June), Summer - (July, August, September), Fall - (October, November, December)) for ecoregions were prepared by estimating the mean and standard deviation of annual SST, SSS, DO and Chlorophyll-*a* was estimated as a measure of degree variability in annual climatology. We used generalized linear models in R 3.6.2 with a binomial link to examine variations in the frequencies of amino acid substitutions under selection (as described by Consuegra *et al*.^45^) in NAS, SEAS, and BoB with SST (Winter, Spring, Summer, and Fall), SSS (Winter, Spring, Summer, and Fall), DO (Spring, Summer, and Fall), Chlorophyll-*a* (Spring, Summer, and Fall), the standard deviation of annual SST (fluctuations in annual SST), the standard deviation of annual SSS (fluctuations in annual SSS), the standard deviation of annual DO (fluctuations in annual DO) and standard deviation of annual Chlorophyll-*a* (fluctuations in Chlorophyll-a).

## Data availability

Mitochondrial DNA sequence data associated with this manuscript have been deposited in GenBank under the accession numbers: MG251937 - MG251981, KJ466087–KJ466091, KJ472113–KJ472120, KJ888156–KJ888390, KP000859–KP000897.

## Supplementary information

Supplemental information.
