# Supplementary material for: Signals of selection in the mitogenome provide insights into adaptation mechanisms in heterogeneous habitats in a widely distributed pelagic fish
Source: Sci Rep. 2020 Jun 3;10:9081. doi: 10.1038/s41598-020-65905-1 (PMC7270097; doi:10.1038/s41598-020-65905-1)
Supplement: Supplementary file 1 — Supplemental information. [file 41598_2020_65905_MOESM1_ESM.pdf]

Signals of selection in the mitogenome provide insights into adaptation mechanisms in heterogeneous habitats in a widely distributed pelagic fish

Wilson Sebastian[1], Sandhya Sukumaran[1*], P.U. Zacharia[1], K. R. Muraleedharan[2], Dinesh Kumar P.K[2]., A. Gopalakrishnan[1]
[1]Marine Biotechnology Division, ICAR-Central Marine Fisheries Research Institute, Ernakulam North P.O., Kochi – 682018, Kerala, India
[2] CSIR-National Institute of Oceanography, Regional Centre Kochi, Dr Salim Ali Road, Post Box No. 1913 Kochi - 682 018, Kerala, India

[*] Corresponding author: Marine Biotechnology Division, Central Marine Fisheries Research Institute, Ernakulam North P.O., Kochi – 682018, Kerala, India
Phone No: 91-9495614119; Email: sukumaransandhya@yahoo.com

Table T1. List of Primer pairs used for amplification of *S. longiceps* mitochondrial DNA

Primer Name		Sequence (5' - 3')	PCR Product length
SPF M 1	Forward primer	AAGAGGGCCGGTAAAACTCG	1080
	Reverse primer	GGTTTCGGGGGCTCAAACTA	
SPF M 2	Forward primer	CACAATATTCGCCGCAAGGG	1140
	Reverse primer	GCGGCCGTTAAACTTTTGGT	
SPF M 3	Forward primer	TCCTGCAGCAAGACATCGTT	1287
	Reverse primer	AGGCTGGATAGGGCCAAAAC	
SPF M 4	Forward primer	GTTTTGGCCCTATCCAGCCT	1390
	Reverse primer	TTGGGTCTGGTTAAGACCGC	
SPF M 5	Forward primer	CCACCCCTACCTCCTAACGA	1267
	Reverse primer	ATGCCATATCAGGTGCTCCG	
SPF M 6	Forward primer	CTCTGTCAGGCAATCTGGCA	1299
	Reverse primer	ACGCAGGGGTTTAACCTACG	
SPF M 7	Forward primer	CGTAGGTTAAACCCCTGCGT	1307
	Reverse primer	AATCACCGTAGCAAGCCACA	
SPF M 8	Forward primer	TGTGGCTTGCTACGGTGATT	1071
	Reverse primer	GCTGCCTCAAACCCAAAGTG	
SPF M 9	Forward primer	ACCACTTTGGGTTTGAGGCA	1130
	Reverse primer	CATGTGGTTCTGGCTGGCTA	
SPF M 10	Forward primer	GATCATCGCCTCTCTGAGCC	1131
	Reverse primer	AGAGAGTACCCGGCTGTGAT	
SPF M11	Forward primer	ATCACAGCCGGGTACTCTCT	1458
	Reverse primer	TTGCTCATCGTTGAGGCTGT	
SPF M 12	Forward primer	ACAGGCACCCCTTTCTTAGC	1344
	Reverse primer	TCTGGAGCTTGTTGCGTCAT	
SPF M 13	Forward primer	AGAGCTCACCGGGTATTCCT	1018
	Reverse primer	AAGTGGAACGCGAAAAACCG	
SPF M 14	Forward primer	CGGTTTTTCGCGTTCCACTT	1287
	Reverse primer	AAGGACTCGCCAGATGCAAA	

Table T2. Sampling locations of *S. longiceps* populations from 3 ecoregions in the Indian Ocean.

Sampling location		Latitude	Longitude
NAS n = 117 (15 Complete mtDNA, 117 control region)	OMAN	19.482°N	63.744°E
	VERAVAL	20.793°N	69.842°E
	MUMBAI	19.465°N	72.111°E
SEAS n = 117 (15 Complete mtDNA, 117 control region)	MANGALURU	13.112°N	74.262°E
	KOZHIKODE	11.101°N	75.251°E
	KOLLAM	9.344°N	76.112°E
	THIRUVANANTHAPURAM	8.182°N	76.933°E
BoB n = 116 (15 Complete mtDNA, 116 control region)	CHENNAI	13.291°N	18.794°E
	VISAKHAPATNAM	17.934°N	84.182°E

NAS (Northern Arabian Sea), SEAS (South Eastern Arabian Sea) and BoB (Bay of Bengal), n number of individuals collected.

Table T3. Nucleotide diversity of *S. longiceps* populations from 3 ecoregions in the Indian Ocean.

	NAS	SEAS	BoB
ND1	0.0064	0.00794	0.0065
ND2	0.00673	0.00878	0.0065
CO1	0.00275	0.00294	0.0021
CO2	0.00279	0.00706	0.0025
ATP8	0.0008	0.00163	0
ATP6	0.00208	0.00606	0.0069
CO3	0.00161	0.00459	0.0073
ND3	0.00172	0.00546	0.00507
ND4L	0.00142	0.00089	0.00112
ND4	0.00639	0.00862	0.00809
ND5	0.00722	0.00878	0.00842
ND6	0.0077	0.00457	0.00696
CYTB	0.0021	0.00488	0.00543
CONTROL	0.0132	0.018	0.01292
ALLGENE	0.0049	0.01678	0.00284
ALLSEQ	0.0045	0.00605	0.00512

NAS (Northern Arabian Sea), SEAS (South Eastern Arabian Sea) and BoB (Bay of Bengal).

Table T4. Amino acid diversity of *S. longiceps* populations from 3 ecoregions in the Indian Ocean.

	NAS	SEAS	BoB
ND1	0	0.000308	0.00414
ND2	0.000703	0.00182	0.00048
ND3	0.001163	0.00204	0
ND4L	0	0	0
ND4	0.00467	0.00382	0.00217
ND5	0.00249	0.00505	0.00414
ND6	0	0.000772	0
CO1	0.00194	0.00203	0.00064
CO2	0.00122	0.00849	0.00145
CO3	0.000423	0.0219	0.00837
ATP8	0	0	0
ATP6	0.00117	0.00563	0.0066
CYTB	0.00434	0.001706	0.00114

NAS (Northern Arabian Sea), SEAS (South Eastern Arabian Sea) and BoB (Bay of Bengal).

Table T5. Seasonal Climatology of 3 ecoregions, Codons that are under positive selection in the mitogenome protein coding genes, and the number of repeat units in control region of *S. longiceps* populations from 3 ecoregions in the Indian Ocean.

Seasonal Climatology						Length polymorphisms in the control region	Codons that are under positive selection in the mitogenome protein coding genes												
Ecoregions	Season	Sea surface temperature (SST) ^{0}C	Sea surface salinity (SSS) ppt	Disssolved oxygen (DO) mg/L	Chlrophyll a mg/m^3	Type 1 with one repeat unit, Type 2 with two repeat unit, Type 3 with three repeat unit,	Complex I (12 sites)							Complex IV (8 sites)			Complex V (2 sites)		Complex I I I (4 sites)
							ND1 (2 sites)	ND2 (1 sites)	ND3	ND4L	ND4 (1 sites)	ND5 (8 sites)	ND6	CO1 (3 sites)	CO2 (3 sites)	CO3 (2 sites)	ATP8	ATP6 (2 sites)	CYTB (4 sites)
NAS	Winter (JFM)	24.0-27.0	36.0-38.0	-	-	Type 1<br>Type 2	-	#302 Leu-Gln	-	-	-	#226Thr-Asn	-	#114 Gly-Ala<br>#262 Asn-Asp	#50 Leu-Gln	-	-	-	#70 Cys-Tyr
	Spring (AMJ)	23.0-25.0	36.5-38.0	3.7-4.0	2.0-10.0														
	Summer (JAS)	20.0-22.5	37-38	1.25-2.75	4.0-10.0														
	Fall (OND)	21.0-22.5	36.5-38.0	2.5-2.75	2.0-5.0														
SEAS	Winter (JFM)	28.0-28.5	33.0-35.0	-	-	Type 1<br>Type 2<br>Type 3	#29 Ile-Phe<br>#30 Glu-Leu	#302 Leu-Gln	-	-	#148 Thr-Asn	#97Ala-Gly<br>#98Leu-Val<br>#225Ala-Thr<br>#226Thr-Asn<br>#227Gly-Cys<br>#228Lys-Asn<br>#236 Pro-Ser	-	#25 Leu-Arg<br>#114 Gly-Ala<br>#262 Asn-Asp	#152 Val-Ser	#16 Trp-Gly, Leu<br>#117 Pro-Leu	-	#114 Val-Leu	#70 Cys-Tyr, Trp
	Spring (AMJ)	29.0-30.0	34.5	3.25-4.25	1.0-5.0														
	Summer (JAS)	26.0-28.0	34.5	1.0-3.25	5.0-10.0														
	Fall (OND)	28.0-29.0	34.5	1.0-1.25	2.0-3.0														
BoB	Winter (JFM)	26.0-27.0	31.5-33.0	-	-	Type 1	-	-	-	-	-	#225 Ala-Thr<br>#227 Gly-Cys<br>#228 Lys-Asn<br>#236 Pro-Phe	-	#262 Asn-Asp	#63 Glu-Gly	#16 Trp-Arg<br>#117 Pro-Ser	-	#114 Val-Ala	#70 Cys-Tyr, Trp
	Spring (AMJ)	29.0-30.0	31.5-33.0	4-5	0.0-2.0														
	Summer (JAS)	28.5-30.0	29.5-33.0	3.0-4.25	1.0-3.0														
	Fall (OND)	26.5-27.5	32.0-28.5	3.25-4.25	0.0-2.0														

NAS (Northern Arabian Sea), SEAS (South Eastern Arabian Sea) and BoB (Bay of Bengal).

Table T6. Codons that are under purifying selection in the mitogenome protein coding genes of *S. longiceps*.

NADH dehydrogenase subunits 1 (ND1) No of sites 79		NADH dehydrogenase subunits 2 (ND2) No of sites 94		Cytochrome c oxidase subunits 1 (COX1) No of sites 58		Cytochrome c oxidase subunits 2 (COX2) No of sites 23		ATPase subunits 8 (ATP8) No of sites 3		ATPase subunits 6 (APT6) No of sites 32		Cytochrome c oxidase subunits 3 (COX3) No of sites 28		NADH dehydrogenase subunits 3 (ND3) No of sites 13		NADH dehydrogenase subunits 4L (ND4L) No of sites 9		NADH dehydrogenase subunits 4 (ND4) No of sites 101		NADH dehydrogenase subunits 5 (ND5) No of sites 143		NADH dehydrogenase subunits 6 (ND6) No of sites 25		Cytochrome b (CYTB), No of sites 73	
codon	Posterior Prob β<α	codon	Posterior Prob β<α	codon	Posterior Prob β<α	codon	Posterior Prob β<α	codon	Posterior Prob β<α	codon	Posterior Prob β<α	codon	Posterior Prob β<α	codon	Posterior Prob β<α	codon	Posterior Prob β<α	codon	Posterior Prob β<α	codon	Posterior Prob β<α	codon	Posterior Prob β<α	codon	Posterior Prob β<α
291	0.935114	27	0.935	337	0.934	145	0.944	6	0.96	67	0.94	223	0.94	90	0.94	60	0.96	150	0.94	376	0.94	114	1	180	1
294	0.935412	295	0.935	129	0.937	141	0.949	11	0.96	178	0.94	120	0.94	103	0.95	43	0.96	328	0.94	214	0.94	157	1	100	1
26	0.938699	242	0.935	351	0.945	147	0.957	44	0.97	84	0.94	173	0.95	82	0.95	7	0.96	271	0.94	496	0.94	144	1	246	0.999
228	0.943783	324	0.944	224	0.946	185	0.961			134	0.94	21	0.95	39	0.95	59	0.96	43	0.94	29	0.94	166	1	105	0.999
87	0.944438	287	0.944	25	0.947	160	0.962			146	0.94	134	0.95	113	0.96	41	0.96	174	0.94	24	0.94	174	1	282	0.999
209	0.946838	107	0.944	328	0.947	143	0.962			97	0.95	202	0.96	32	0.96	88	0.97	115	0.94	135	0.94	164	1	251	0.999
308	0.947344	81	0.947	232	0.948	62	0.965			106	0.95	52	0.96	38	0.97	48	0.97	23	0.94	270	0.94	147	0.99	165	0.998
66	0.950452	113	0.947	128	0.951	133	0.965			65	0.95	169	0.96	89	0.98	45	0.99	443	0.94	323	0.94	142	0.99	55	0.998
73	0.951221	162	0.948	175	0.951	198	0.966			121	0.95	185	0.96	110	0.98	2	1	408	0.94	227	0.94	118	0.99	120	0.998
153	0.957453	114	0.948	436	0.951	108	0.966			82	0.96	67	0.96	79	0.99			292	0.94	479	0.94	126	0.99	369	0.997
126	0.957512	320	0.949	316	0.955	211	0.967			166	0.96	140	0.96	80	0.99			189	0.95	150	0.95	156	0.99	113	0.994
222	0.957603	173	0.949	270	0.957	174	0.969			88	0.96	239	0.96	41	1			88	0.95	167	0.95	130	0.97	159	0.994
95	0.957955	341	0.951	144	0.957	119	0.969			138	0.96	144	0.97	28	1			190	0.95	410	0.95	173	0.97	361	0.994
137	0.958009	223	0.951	413	0.957	146	0.97			20	0.96	116	0.97					360	0.95	271	0.95	143	0.97	127	0.993
28	0.958023	120	0.957	75	0.958	36	0.971			116	0.96	174	0.97					255	0.95	306	0.95	125	0.97	275	0.993
108	0.959467	277	0.957	267	0.958	57	0.971			64	0.96	201	0.97					347	0.95	567	0.95	170	0.97	115	0.993
183	0.960141	167	0.958	192	0.958	70	0.973			34	0.96	161	0.97					163	0.96	209	0.95	152	0.97	337	0.993
212	0.960159	256	0.958	195	0.958	167	0.974			14	0.96	117	0.97					194	0.96	535	0.95	167	0.97	147	0.992
255	0.960179	132	0.959	261	0.958	59	0.988			111	0.97	198	0.97					171	0.96	109	0.95	104	0.96	299	0.992
314	0.960179	78	0.959	379	0.959	105	0.991			60	0.97	258	0.98					420	0.96	315	0.95	172	0.96	194	0.991
96	0.96034	84	0.96	335	0.96	203	0.998			71	0.97	23	0.99					63	0.96	91	0.95	133	0.96	141	0.99
284	0.961381	119	0.961	171	0.961	137	0.998			110	0.97	136	0.99					349	0.96	524	0.95	127	0.96	81	0.989
272	0.963232	272	0.961	482	0.961	148	0.999			29	0.97	162	0.99					314	0.96	498	0.95	100	0.96	128	0.986
91	0.963504	181	0.961	305	0.961					171	0.97	147	0.99					202	0.96	241	0.95	150	0.96	213	0.984
239	0.96355	294	0.961	213	0.963					152	0.97	123	0.99					366	0.96	565	0.96	119	0.96	326	0.981
166	0.963688	60	0.964	102	0.964					55	0.98	70	1					440	0.96	46	0.96			266	0.979
99	0.96391	259	0.964	392	0.964					135	0.99	93	1					425	0.96	191	0.96			109	0.978
7	0.964355	229	0.964	414	0.964					164	0.99	64	1					19	0.96	355	0.96			293	0.978
142	0.964745	58	0.964	317	0.966					63	0.99							81	0.96	612	0.96			258	0.974
22	0.966039	176	0.964	209	0.966					15	1							311	0.96	460	0.96			156	0.971
172	0.966459	24	0.965	314	0.966					208	1							164	0.96	53	0.96			237	0.971
136	0.968353	280	0.965	2	0.967					204	1							343	0.96	399	0.96			256	0.97
193	0.968412	71	0.965	349	0.967													422	0.96	389	0.96			30	0.97
33	0.968926	319	0.965	373	0.968													184	0.96	154	0.96			252	0.97
315	0.968926	225	0.965	255	0.968													38	0.96	324	0.96			347	0.969
229	0.969505	237	0.965	474	0.968													415	0.96	198	0.96			95	0.969
236	0.969822	275	0.966	141	0.969													26	0.96	470	0.96			260	0.969
14	0.970518	257	0.966	319	0.969													134	0.97	245	0.96			228	0.969
24	0.970518	332	0.966	333	0.97													298	0.97	122	0.96			119	0.969
238	0.972163	108	0.966	339	0.971													132	0.97	260	0.96			288	0.968
11	0.974071	95	0.966	14	0.972													339	0.97	332	0.96			306	0.968
200	0.974132	131	0.966	97	0.974													281	0.97	381	0.96			44	0.967
216	0.979368	307	0.966	470	0.978													283	0.97	126	0.96			151	0.966
296	0.979446	172	0.966	200	0.978													414	0.97	452	0.96			178	0.966
109	0.980475	282	0.966	456	0.981													41	0.97	531	0.96			297	0.966
69	0.980659	145	0.967	396	0.982													309	0.97	451	0.96			196	0.966
290	0.981118	179	0.967	505	0.986													83	0.97	433	0.96			212	0.966
285	0.982982	157	0.967	211	0.989													371	0.97	105	0.96			231	0.966
60	0.983604	77	0.968	355	0.991													140	0.97	275	0.96			33	0.965
102	0.983826	85	0.968	189	0.991													188	0.97	106	0.96			160	0.965
268	0.985148	208	0.968	480	0.993													18	0.97	326	0.96			122	0.964

114	0.985734	69	0.968	205	0.994													405	0.97	102	0.96			204	0.964
198	0.986421	92	0.968	422	0.994													66	0.97	311	0.97			248	0.963
164	0.987846	329	0.969	350	0.994													381	0.97	485	0.97			270	0.962
206	0.988031	292	0.969	123	0.995													441	0.97	583	0.97			322	0.961
135	0.991138	268	0.969	203	0.998													121	0.97	284	0.97			314	0.961
143	0.991212	192	0.969	401	0.999													312	0.97	390	0.97			245	0.961
233	0.992462	200	0.971	387	0.999													91	0.97	143	0.97			155	0.961
149	0.992889	300	0.971															240	0.97	409	0.97			28	0.96
176	0.993387	121	0.973															141	0.97	100	0.97			190	0.96
50	0.993754	128	0.973															430	0.97	96	0.97			117	0.959
92	0.994485	343	0.978															389	0.97	107	0.97			145	0.959
243	0.994657	233	0.98															388	0.97	371	0.97			129	0.959
185	0.994897	146	0.986															320	0.97	196	0.97			255	0.958
263	0.995027	199	0.986															55	0.98	272	0.97			161	0.957
16	0.995153	168	0.988															456	0.98	228	0.97			46	0.957
292	0.996801	283	0.988															274	0.98	88	0.97			316	0.95
168	0.997038	337	0.99															331	0.98	162	0.97			356	0.949
259	0.997266	303	0.99															304	0.98	325	0.97			195	0.947
287	0.99816	227	0.991															120	0.98	220	0.97			307	0.944
248	0.998406	211	0.991															178	0.98	62	0.97			253	0.944
174	0.998501	297	0.991															162	0.98	351	0.97			126	0.944
215	0.99866	64	0.991															323	0.99	43	0.97			279	0.935
46	0.998967	74	0.992															183	0.99	117	0.97				
191	0.999381	104	0.992															94	0.99	331	0.97				
163	0.999707	90	0.993															387	0.99	553	0.97				
18	0.999932	253	0.993															85	0.99	281	0.97				
122	0.999986	308	0.994															11	0.99	456	0.97				
165	0.999993	56	0.994															336	0.99	571	0.97				
		125	0.994															181	0.99	181	0.97				
		45	0.994															102	0.99	278	0.97				
		180	0.994															170	0.99	305	0.97				
		139	0.994															231	0.99	296	0.97				
		163	0.996															125	0.99	412	0.97				
		129	0.996															60	1	577	0.97				
		54	0.996															34	1	476	0.97				
		210	0.997															383	1	539	0.97				
		239	0.997															384	1	193	0.97				
		164	0.998															452	1	165	0.97				
		143	0.999															380	1	386	0.97				
		298	0.999															365	1	328	0.97				
		150	1															52	1	202	0.97				
		59	1															247	1	121	0.97				
		116	1															159	1	204	0.97				
																		259	1	533	0.97				
																		299	1	366	0.97				
																		315	1	224	0.98				
																		288	1	352	0.98				
																		44	1	525	0.98				
																		437	1	180	0.98				
																		59	1	382	0.98				
																				112	0.99				
																				141	0.99				
																				609	0.99				
																				276	0.99				
																				277	0.99				
																				279	0.99				
																				36	0.99				
																				550	0.99				
																				183	0.99				
																				443	0.99				
																				566	0.99				
																				463	0.99				
																				142	0.99				

																				213	0.99				
																				482	1				
																				551	1				
																				584	1				
																				572	1				
																				208	1				
																				422	1				
																				273	1				
																				395	1				
																				166	1				
																				429	1				
																				94	1				
																				203	1				
																				147	1				
																				589	1				
																				327	1				
																				339	1				
																				500	1				
																				595	1				
																				491	1				
																				108	1				
																				185	1				
																				188	1				
																				449	1				
																				557	1				
																				514	1				
																				486	1				
																				189	1				

The analysis is performed in FUBAR at posterior probability ≥ 0.9.

Table T7. Free energy, ΔG for 22 *S. longiceps* mitochondrial tRNA genes and predicted secondary structures of repeat unit types (Figure A10).

tRNA (RNA)	ΔG(kcal/mol)	Length(bp)	Normalized free energy -ΔG(kcal/mol)/ Length(bp)
tRNA-Ala	-10.77	69	-0.156
tRNA-Arg	-16.3	69	-0.236
tRNA-Asn	-10.12	73	-0.139
tRNA-Asp	-10.37	69	-0.150
tRNA-Cys	-21.7	66	-0.329
tRNA-Gln	-16.21	71	-0.228
tRNA-Glu	-6.1	69	-0.088
tRNA-Gly	-20.3	71	-0.286
tRNA-His	-14.6	69	-0.212
tRNA-Ile	-30.31	72	-0.421
tRNA-Leu	-20.5	75	-0.273
tRNA-Leu	-27.4	72	-0.381
tRNA-Lys	-19.6	74	-0.265
tRNA-Met	-16.24	69	-0.235
tRNA-Phe	-12.34	63	-0.196
tRNA-Pro	-17.1	70	-0.244
tRNA-Ser	-19.2	68	-0.282
tRNA-Ser	-11.31	67	-0.169
tRNA-Thr	-28.2	72	-0.392
tRNA-Trp	-9.07	70	-0.130
tRNA-Tyr	-15.96	71	-0.225
tRNA-Val	-20.3	72	-0.282
Repeat unit predicted structure	ΔG(kcal/mol)	Length(bp)	
Type 1 DNA	-8.79	67	-0.131
Type 1 RNA	-17.4	67	-0.260
Type 2 DNA	-24.17	108	-0.224
Type 2 RNA	-41.9	108	-0.388
Type 3a_1 DNA	-15.43	147	-0.105
Type 3a_1 RNA	-39	147	-0.265
Type 3a_2 DNA	-15.43	147	-0.105
Type 3a_2 RNA	-39.5	147	-0.269
Type 3a_3 DNA	-15.22	147	-0.104
Type 3a_4 DNA	-15.21	147	-0.103
Type 3a_5 DNA	-14.97	147	-0.102
Type 3b_1 DNA	-16.64	143	-0.116
Type 3b_1 RNA	-43.9	143	-0.307
Type 3b_2 DNA	-16.42	143	-0.115
Type 3b_3 DNA	-15.87	143	-0.111
Type 3b_4 DNA	-15.65	143	-0.109
Type 3c_1 DNA	-26.24	147	-0.179
Type 3c_1 RNA	-49.4	147	-0.336
Type 3c_2 RNA	-47.5	147	-0.323
Type 3d_1 DNA	-16.05	147	-0.109
Type 3d_5 DNA	-15.23	147	-0.104
Type 3d_1 RNA	-38.65	147	-0.263
Type 3d_2 DNA	-15.73	147	-0.107
Type 3d_2 RNA	-39.5	147	-0.269
Type 3d_3 DNA	-15.58	147	-0.106
Type 3d_4 DNA	-15.35	147	-0.104

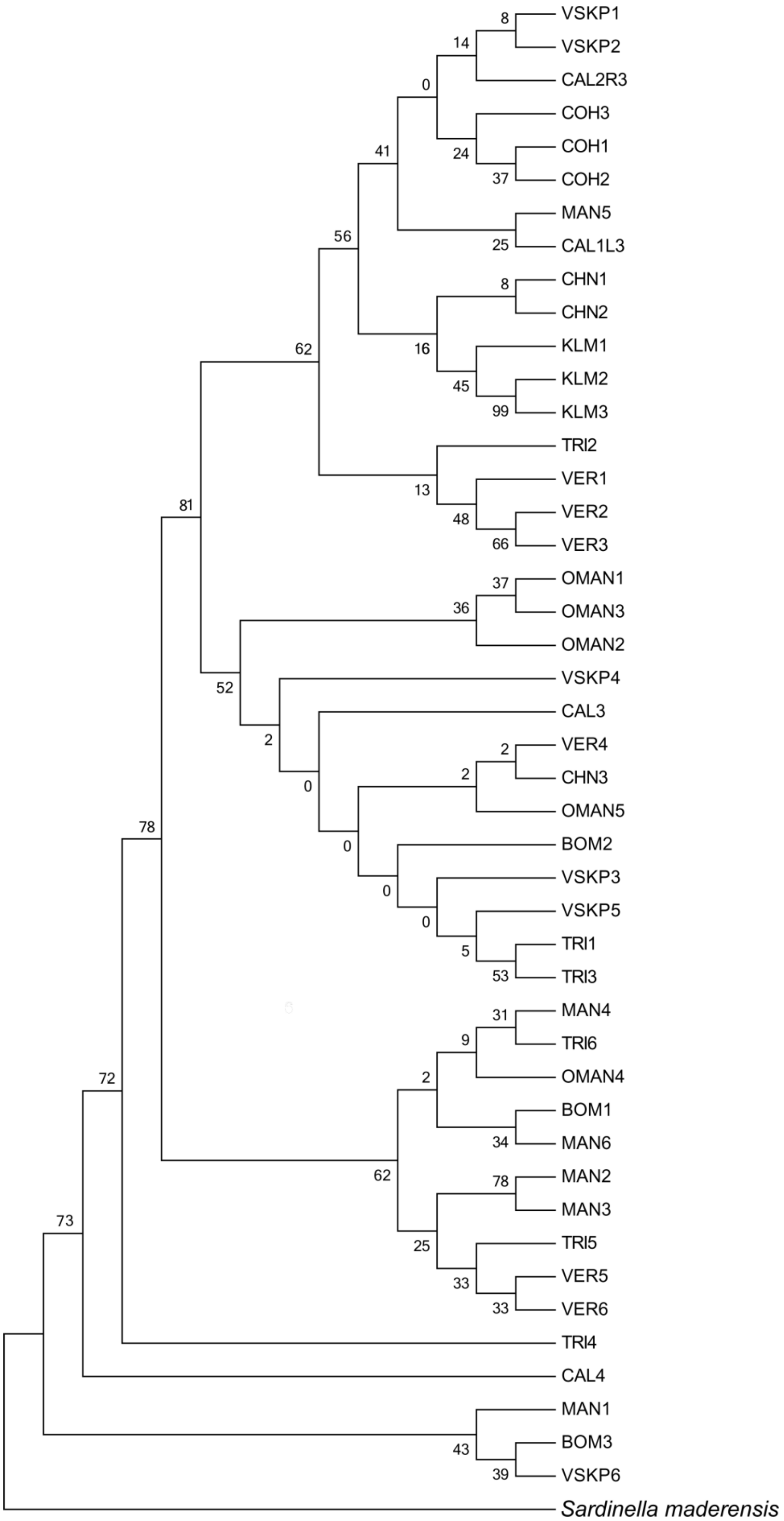

Figure A1. Maximum likelihood tree for whole mitogenome nucleotide sequences of *S.longiceps.* Bootstrap values for node support are shown. *Sardinella maderensis* (Gen Bank accession number NC009587) was used as an outgroup to root the tree.

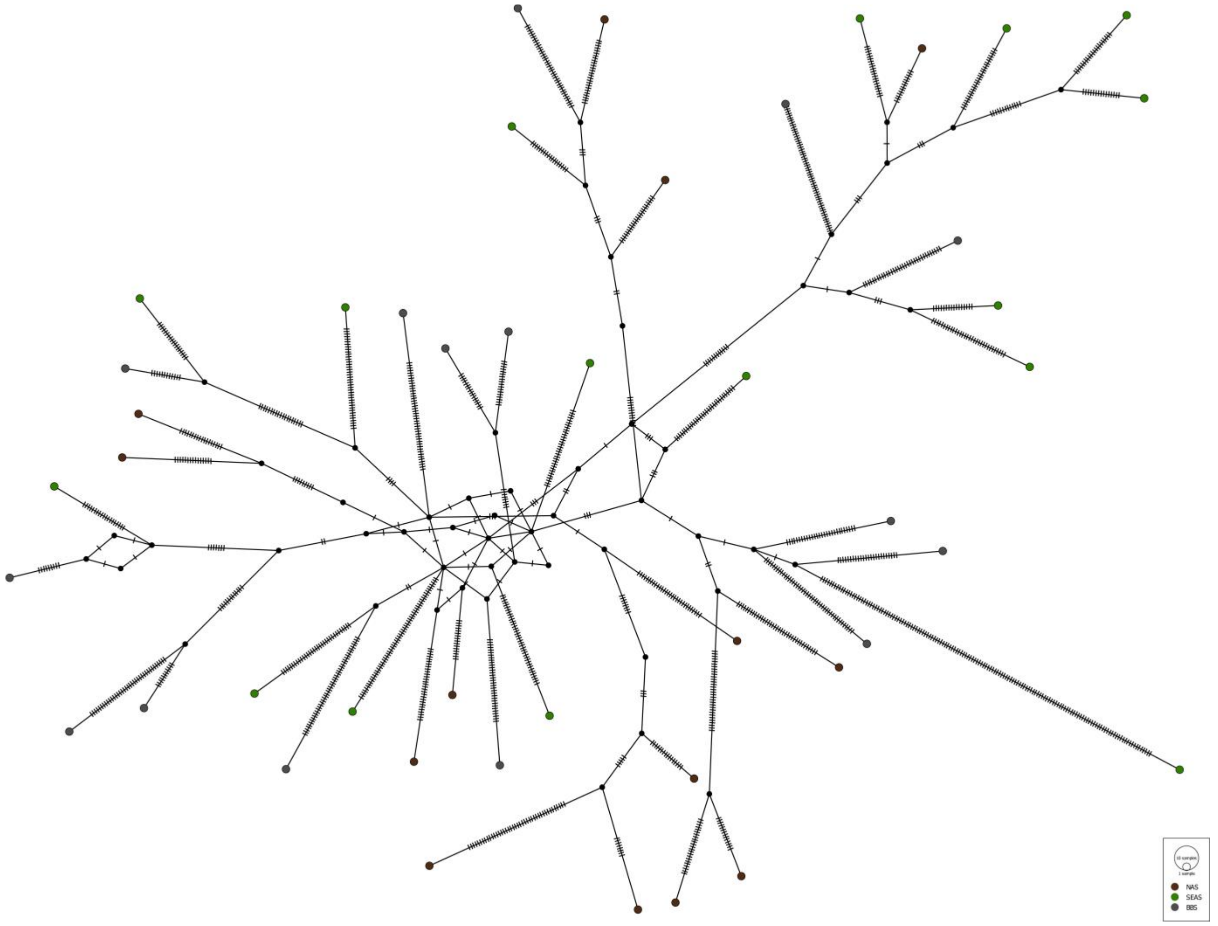

Figure A2. Median joining haplotype network tree of whole mito-genome sequences of 45 *S. longiceps*. Haplotypes are represented in circles and mutational steps are indicated as hatch marks.

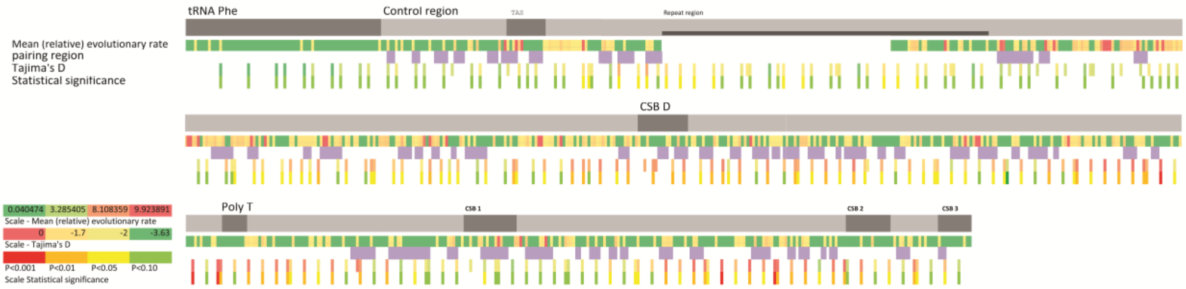

Figure. A3. Schematic representation of the *S. longiceps* mtDNA region of ~1112bp comprising tRNA pro control region and tRNA phe. The locations of the two tRNA coding flanking regions are indicated in black color. The characteristic sequence block in control region are indicated as CSBs - conserved blocks, TAS - Termination associated sequence and Poly T. the repeat region between TAS and Poly T is indicated as black line. Mean (relative) evolutionary rate are shown for each base pair below the site. These rates are scaled such that the average evolutonary rate across all sites is 1. This means that sites showing a rate < 1 are evolving slower than average. Tajima's D and its Statistical significance of ~1112bp comprising tRNA pro control region and tRNA phe with 10bp intervals overlapping at 5bp are shown.

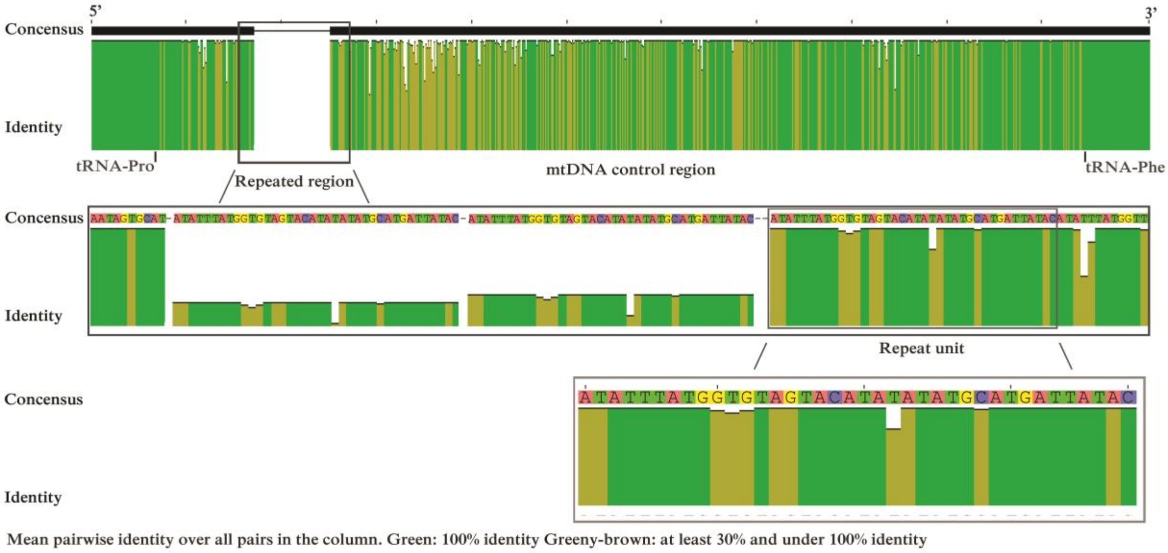

Figure A4. Schematic representation of the *S. longiceps* mtDNA region of ~1112bp comprising tRNA pro control region and tRNA phe. The locations of the two tRNA coding flanking regions are indicated in black color. The characteristic sequence block in control region are indicated as CSBs - conserved blocks, TAS - Termination associated sequence and Poly T. the repeat region between TAS and Poly T is indicated as black line. Mean (relative) evolutionary rate are shown for each base pair below the site. These rates are scaled such that the average evolutonary rate across all sites is 1. This means that sites showing a rate < 1 are evolving slower than average. Tajima's D and its Statistical significance of ~1112bp comprising tRNA pro control region and tRNA phe with 10bp intervals overlapping at 5bp are shown.

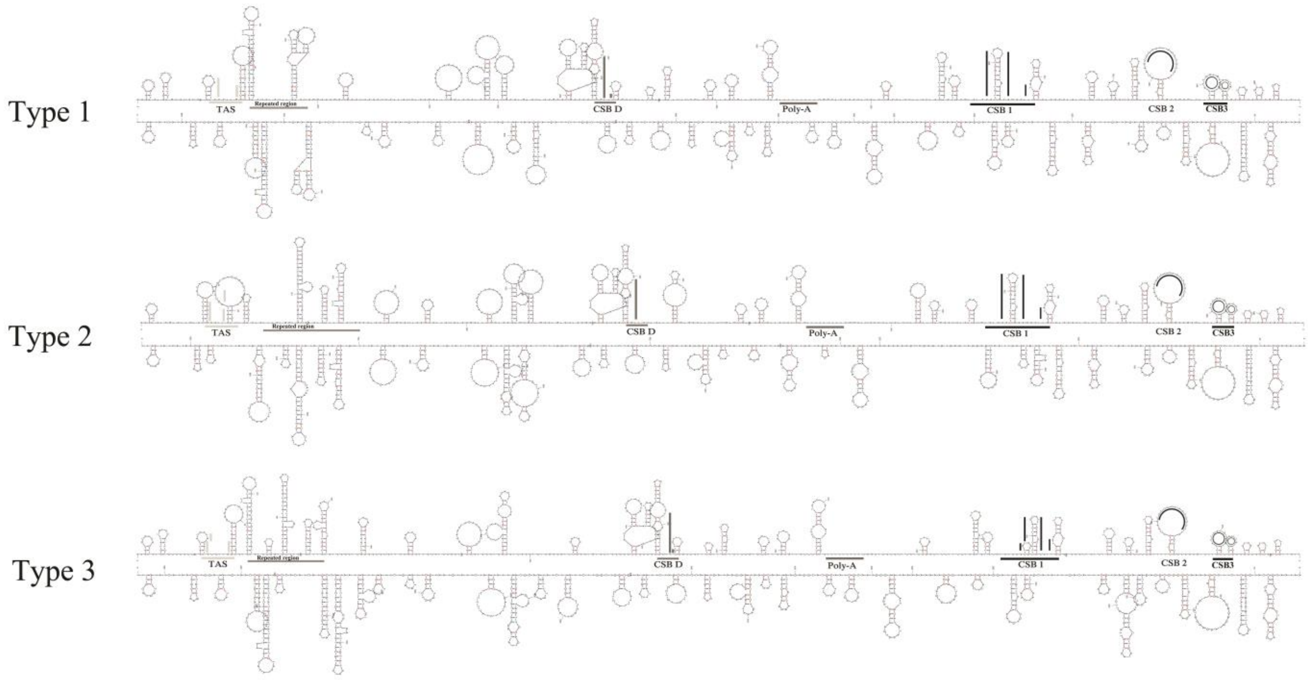

Figure A5. Secondary structures identified in the mtDNA control region of *S. longiceps.*mtDNA control region haplotype with Type 1, 2 and 3 repeat units are indicated as Type 1, Type 2 and Type 3 respectively.

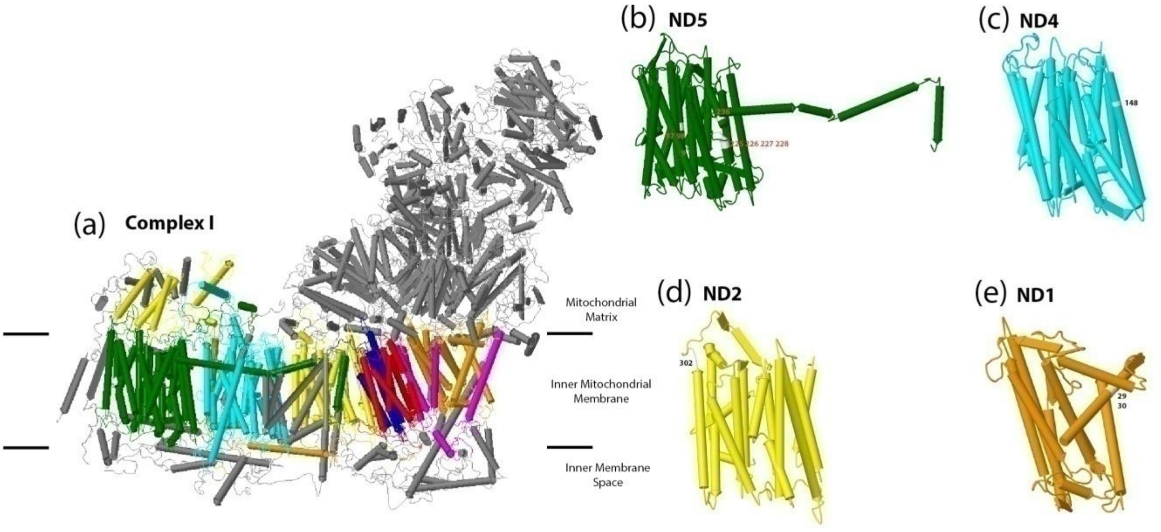

Figure A6. Spatial distribution of positive selected sites identified in NADH dehydrogenase (Complex I) of *S. longiceps*. Grey structures represent nuclear-encoded subunits. (a) individual OXPHOS Complex I, with mitochondrial-encoded subunits are represented in different colored as followed: ND2 in yellow; ND4L in blue; ND1 in orange; ND3 in magenta; ND4 in cyan; ND5 in green; ND6 in red. Individual core subunits (b) ND5, (c) ND4, (d) ND2, (e) ND1with amino acid site number on positively selected sites.

ND1, ND2, ND4 and ND5 sub unit proteins showed 75% identity with Chain H (PDB: 5LDX_H), 50% identity with Chain N (PDB: 5LDX_N), 62% identity with Chain M (PDB: 5LDX_M) and 63% identity with Chain L (PDB: 5LDX_L) respectively of *Bos taurus* Respiratory Complex I (Zhu et al. 2016). Mitochondrial complex I (NADH: ubiquinone oxidoreductase) contributes to cellular energy production by transferring electrons from NADH to ubiquinone coupled to proton translocation across the membrane. The Key polar amino acid residues which have been reported to participate in proton translocation (ND1 - E198, E149, ND2 - K263, K135, K105, E34, ND4 - E124, K238, E379, K208, ND5 -E149, H253, K397) (Zhu et al. 2016) through complex I were conserved in sardine except site 228 in ND5. Site 29ND1, 30ND1, 302ND2, 148ND4, 9ND5, 97ND5, 98ND5, 225ND5, 226ND5, 227ND5, 228ND5 and 236ND5 were identified as positively selected in *S. longiceps* and all of them were located in transmembrane helices except one which is in intra-helix loop (228ND5). Nine of these sites one in ND2 (#302Leu-Gln) were located in C-terminus, one in ND4 (#148Thr-asn) located in proton-conducting membrane transporter (Proton_antipo_M) and seven in ND5 (#97Ala-Gly, #98Leu-Val, #225Ala-Thr, #226Thr-Asn, #227Gly-Cys, #228Lys-Asn & #236Pro-Ser) clustered in Proton_antipo_M & N-terminal (Proton_antipo_N). Position 228 (ND5) showed overlap with amino acid residue that have been reported as one of the key residue in proton translocation.

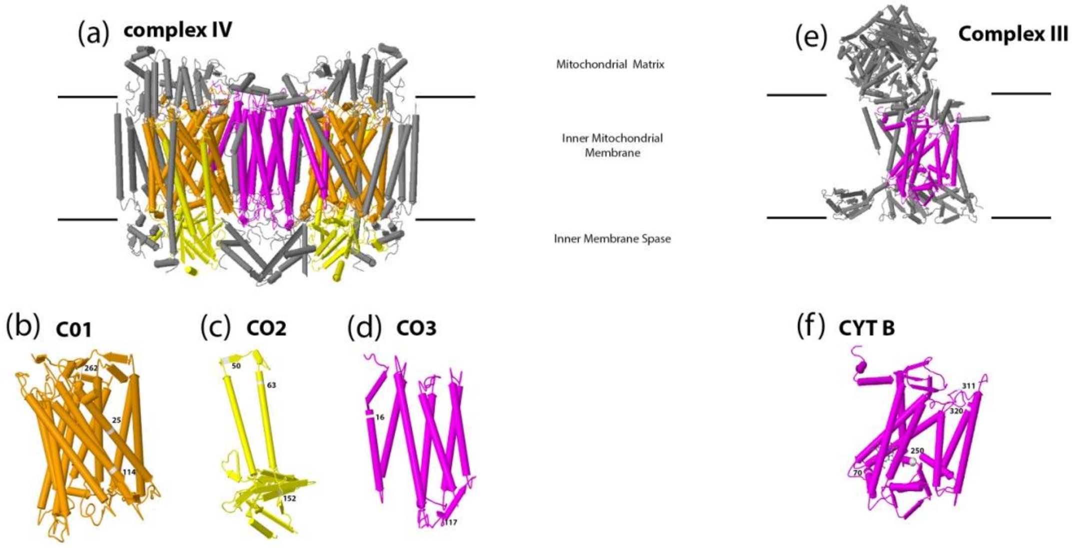

Figure A7. Spatial distribution of positive selected sites identified in Cytochrome C Oxidase (Complex IV) and Cytochrome bc 1 (Complex III) of *S. longiceps*. Grey structures represent nuclear-encoded subunits. (a) Individual OXPHOS Complex IV (Homodimer) with mitochondrial-encoded subunits is represented in different colors as followed: CO1 in orange; CO2 in yellow; CO3 in magenta. (e) Individual OXPHOS Complex III with mitochondrial-encoded subunit represented in magenta colour. Individual core subunits (b) CO1, (c) CO2, (d) CO3, (f) CYT B with amino acid site number at positively selected sites.

CO1, CO2 and CO3 of *S. longiceps* showed 89% identity with Chain N (PDB: 2OCC_N), 73% identity with Chain B (PDB: 2OCC_B) and 80% identity with Chain C (PDB: 2OCC_C) of *B. taurus* Cytochrome C Oxidase (CcO) respectively (Tsukihara et al. 1996). The amino acid residues that have been reported to participate in Electron transfer pathway (F377, R438, R439), D-pathway (Y19, N80, D91, N98, S101, S156, S157, N163, T167), Putative water exit pathway (D227, G232, H233, D364, H368, D369, R438), Ion binding (Binuclear center-heme a3/CuB) (H240, H290, H291, H376), K-pathway (H240, Y244, S255, H290, H291, T316, K319), Putative proton exit pathway (H291, H368, D369, R438, R439), and chemical binding (Low-spin heme a binding site) (H61, H378, S382, T424, S461) in CO1 (Tsukihara et al. 1995) were conserved. Three sites (#25Leu-Arg, #114 Gly-Ala and #262Asn-asp) observed under positive selection in CO1 were located in the transmembrane helix and two of these position (#25 & #114) showed overlap with amino acid residue that have been reported to participate in polypeptide binding at Subunit I/VIIc interface & Subunit I/VIIa interface respectively. Amino acid residues that have been reported to participate in CuA binding site in CO2 and most of the amino acid participated in polypeptide binding & Phospholipid binding in CcO is conserved in *S. longiceps*. Among three sites observed under positive selection in CO2 gene, amino acid position 50 (Leu-gln) reside in the intra-helix loop, position 63 (Glu-gly) in transmembrane helix and 152 (Val-ser) in Beta strand. Among the two sites identified in CO3, position 16 (Trp-Gly) were located in transmembrane helix and position 117 (Pro-Leu,Ser) in the intra-helix loop.

Cytochrome b of *S. longiceps* showed 75% identity with Chain b (PDB: 5LUF_b) of *B. taurus* (Sousa et al. 2016). Majority of amino acid sites that have been suggested to participate in Qo binding, Qi binding and chemical binding were conserved. Among four sites (#70Cys-Trp, #250Leu-Gln, #311 Lys-Glnand #320Leu-Ile) that experienced positive selection in CYTB, one (#311) showed overlap with amino acid residue that have been reported to participate in polypeptide binding in inter-chain domain interface and it was located in the transmembrane helix.

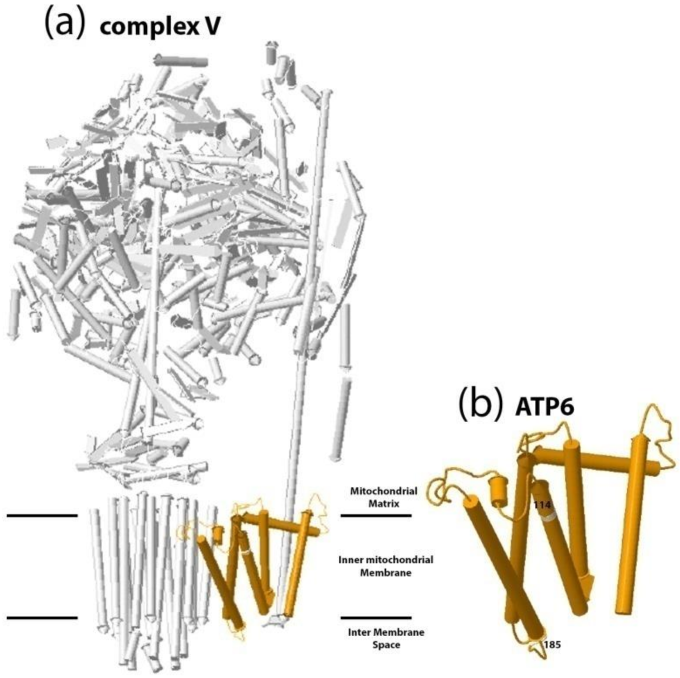

Figure A8. Spatial distribution of positive selected sites identified in ATP synthase (complex V) of *S. longiceps.*Grey structures represent nuclear-encoded subunits. (a) Individual OXPHOS Complex V, with mitochondrial-encoded subunit in orange colour. (b) Individual core subunits ATP 6 with amino acid site number on positively selected sites.

ATP 6 of *S. longiceps* showed 54% identity with Chain W (PDB: 5ARA_W) of *B. taurus* mitochondrial ATP Synthase (Zhou et al. 2015). The highly conserved residue Arg159 in ATP Synthase a subunit showed overlap with Arg at site 160 (middle of helix 5). Among two sites (#114 Val-cys, ala #185 Ile-gln) observed under positive selection, one (#114) was located in the transmembrane helix-4 and other (#185) in intra-helix loop connecting helix-5 and 6.

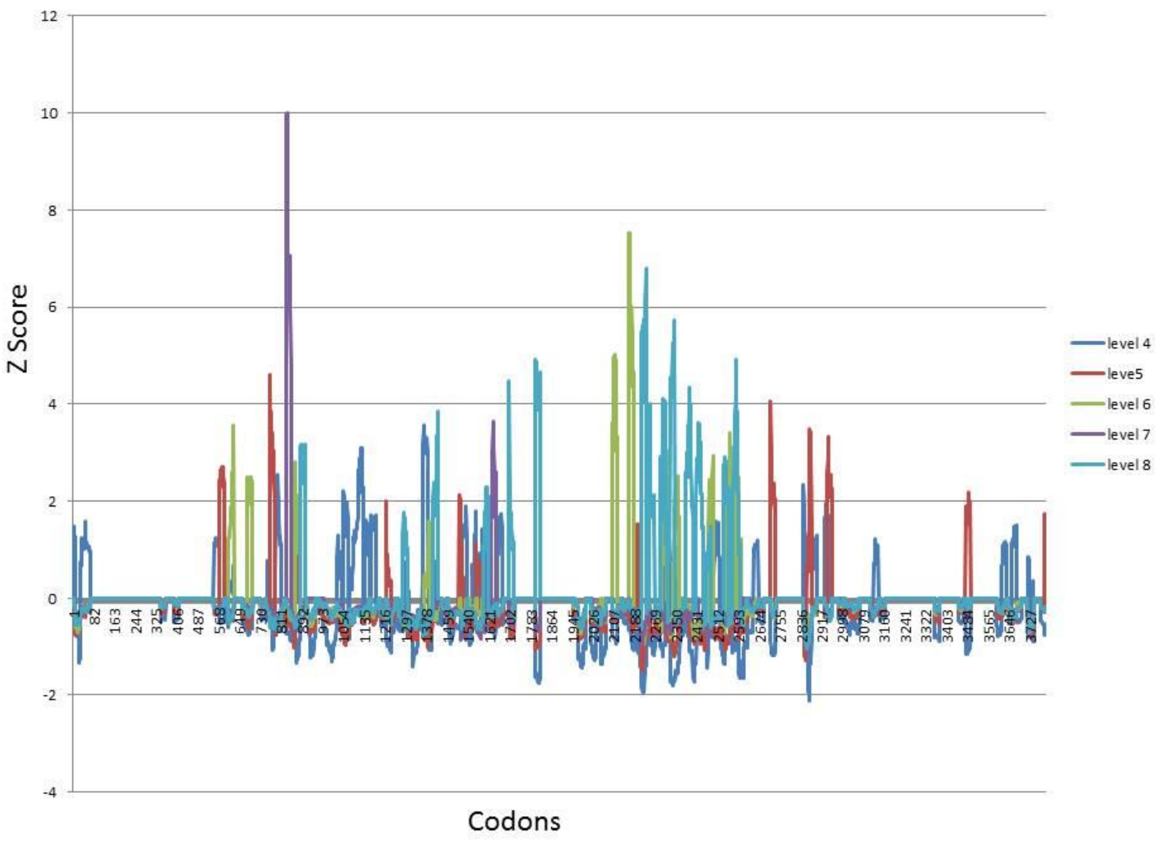

Figure A9. **TreeSAAP results showing region of the mitochondrial genome under positive disruptive selection.** The Z Score shown with horizontal lines, and vertical numerical number indicate amino acid positions in concatenated gene data set. Category of amino acid physiochemical propertychanges are represented as level 4 to level 8. Amino acid position of each individual coding gene in the concatenated gene data set : 1466-1692 ATPase subunits 6 (APT6), 1410-1465 ATPase subunits 8 (ATP8) 675-1179 Cytochrome c oxidase subunits 1 (COX1) 1180-1409 Cytochrome c oxidase subunits 2 (COX2) 1693-1954 Cytochrome c oxidase subunits 3 (COX3) 3416-3795 Cytochrome b (CYTB), 1-325 NADH dehydrogenase subunits 1 (ND1), 326-674 NADH dehydrogenase subunits 2 (ND2), 1955-2070 NADH dehydrogenase subunits 3 (ND3), 2071-2169 NADH dehydrogenase subunits 4L (ND4L), 2170-2629 NADH dehydrogenase subunits 4 (ND4), 2630-3241 NADH dehydrogenase subunits 5 (ND5), 3242-3415 NADH dehydrogenase subunits 6 (ND6).

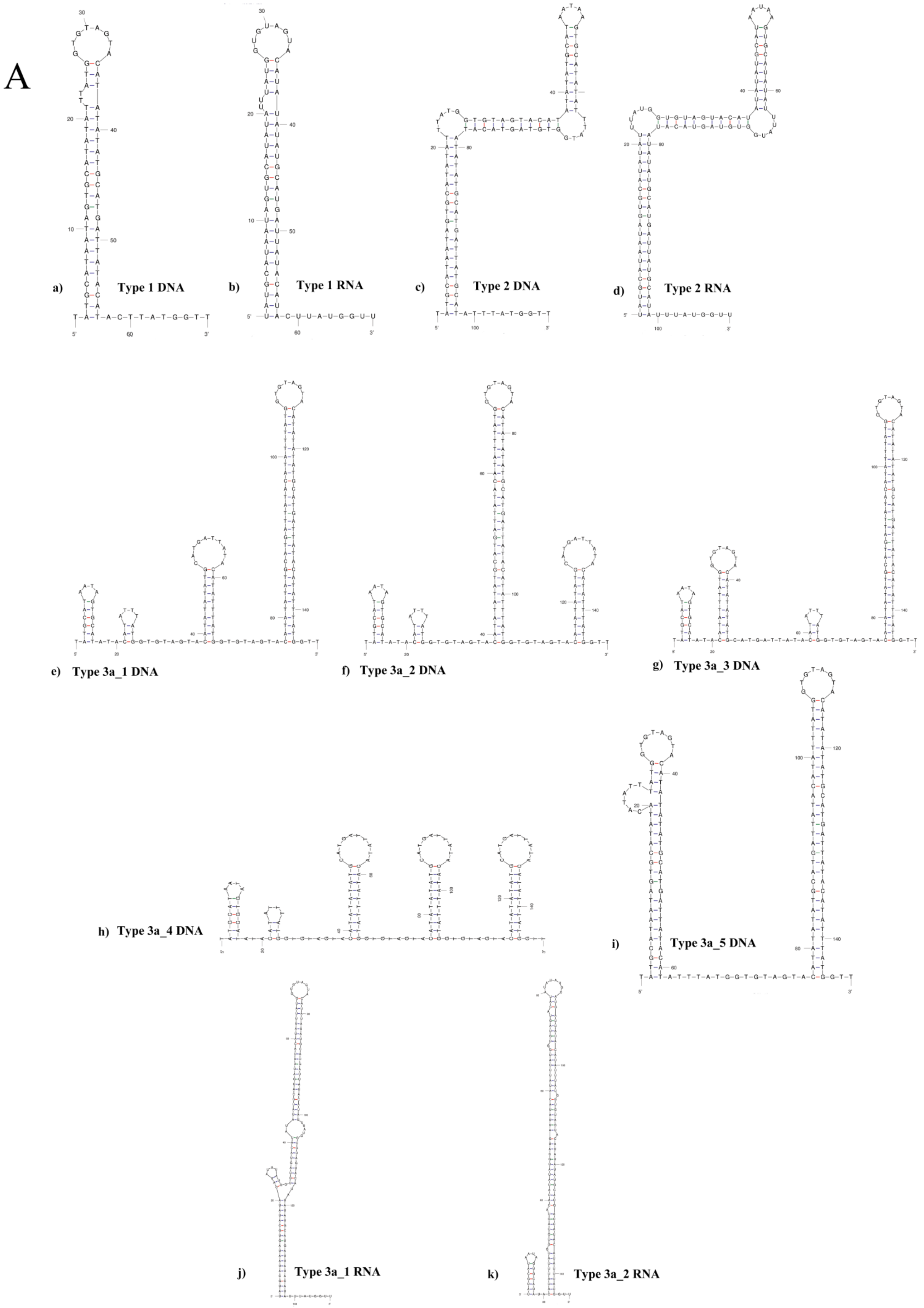
A
a) Type 1 DNA
b) Type 1 RNA
c) Type 2 DNA
d) Type 2 RNA
e) Type 3a_1 DNA
f) Type 3a_2 DNA
g) Type 3a_3 DNA
h) Type 3a_4 DNA
i) Type 3a_5 DNA
j) Type 3a_1 RNA
k) Type 3a_2 RNA

B

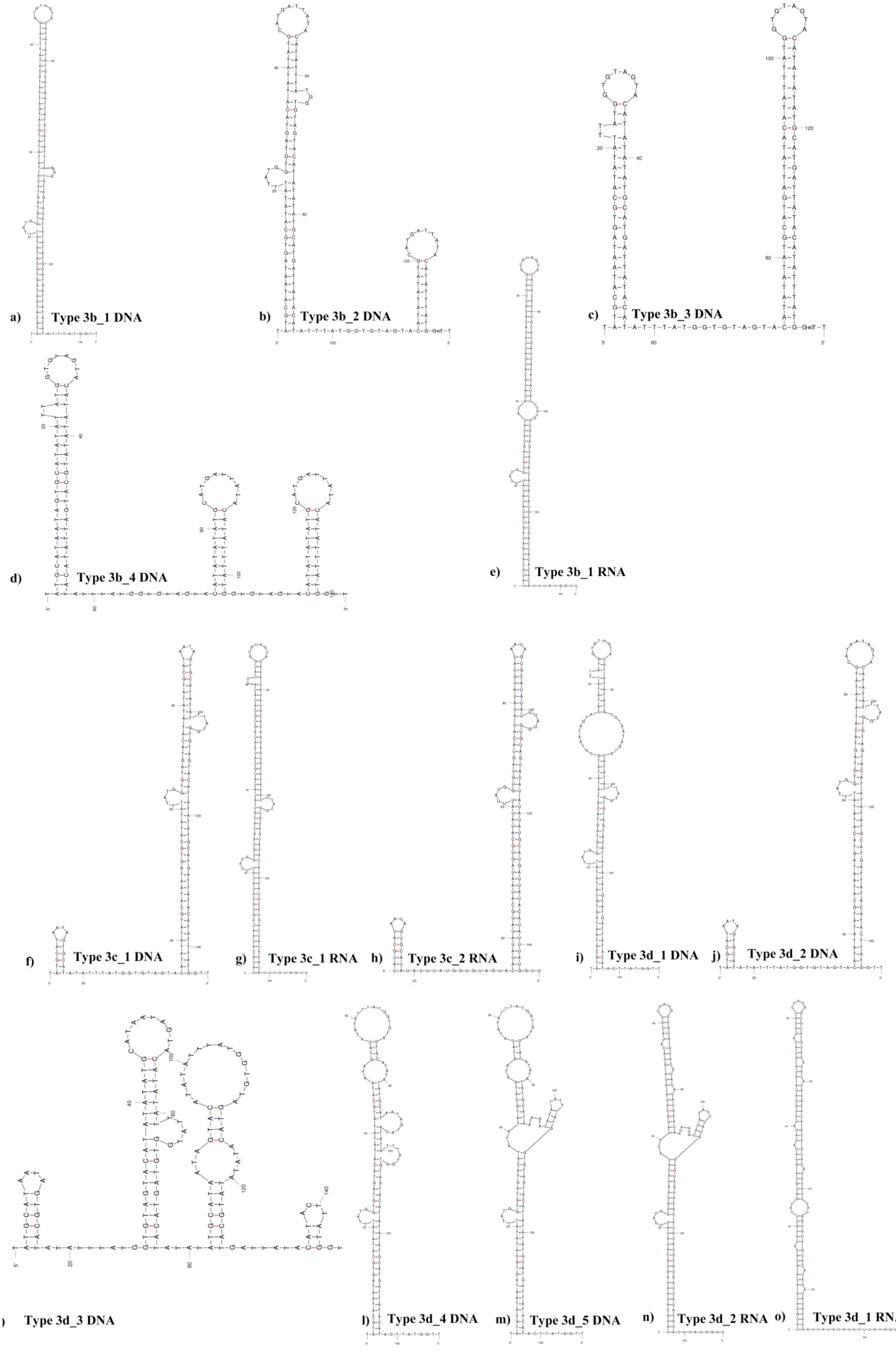
a) Type 3b_1 DNA
b) Type 3b_2 DNA
c) Type 3b_3 DNA
d) Type 3b_4 DNA
e) Type 3b_1 RNA
f) Type 3c_1 DNA
g) Type 3c_1 RNA
h) Type 3c_2 RNA
i) Type 3d_1 DNA
j) Type 3d_2 DNA
) Type 3d_3 DNA
l) Type 3d_4 DNA
m) Type 3d_5 DNA
n) Type 3d_2 RNA
o) Type 3d_1 RNA

Figure A10. Graphical representation of all predicted secondary structures in repeat unit Type 1, 2 and 3 of mtDNA controlregion DNA and the same for RNA. In section A: a) DNA of haplotype with Type 1 repeat unit, b) RNA of haplotype with Type 1 repeat unit, c) DNA of haplotype with Type 2 repeat unit, d) RNA of haplotype with Type 2 repeat unit, e) Structural variant 1 for DNA of haplotype with Type 3 repeat unit variant (Type 3a), f) Structural variant 2 for DNA of haplotype with Type 3 repeat unit variant (Type 3a), g) Structural variant 3 for DNA of haplotype with Type 3 repeat unit variant (Type 3a), h) Structural variant 4 for DNA of haplotype with Type 3 repeat unit variant (Type 3a), i) Structural variant 5 for DNA of haplotype with Type 3 repeat unit variant (Type 3a), j) Structural variant 1 for RNA of haplotype with Type 3 repeat unit variant (Type 3a), k) Structural variant 2 for RNA of haplotype with Type 3 repeat unit variant (Type 3a). In section B: a) Structural variant 1 for DNA of haplotype with Type 3 repeat unit variant (Type 3b), b) Structural variant 2 for DNA of haplotype with Type 3 repeat unit variant (Type 3b), c) Structural variant 3 for DNA of haplotype with Type 3 repeat unit variant (Type 3b), d) Structural variant 4 for DNA of haplotype with Type 3 repeat unit variant (Type 3b), e) Structural variant 1 for RNA of haplotype with Type 3 repeat unit variant (Type 3b), f) Structural variant 2 for DNA of haplotype with Type 3 repeat unit variant (Type 3c), Structural variant 1 for RNA of haplotype with Type 3 repeat unit variant (Type 3c), Structural variant 2 for RNA of haplotype with Type 3 repeat unit variant (Type 3c), Structural variant 1 for DNA of haplotype with Type 3 repeat unit variant (Type 3c), Structural variant 2 for DNA of haplotype with Type 3 repeat unit variant (Type 3c), Structural variant 3 for DNA of haplotype with Type 3 repeat unit variant (Type 3c), Structural variant 4 for DNA of haplotype with Type 3 repeat unit variant (Type 3c), Structural variant 5 for DNA of haplotype with Type 3 repeat unit variant (Type 3c), Structural variant 1 for RNA of haplotype with Type 3 repeat unit variant (Type 3c), Structural variant 2 for RNA of haplotype with Type 3 repeat unit variant (Type 3c).

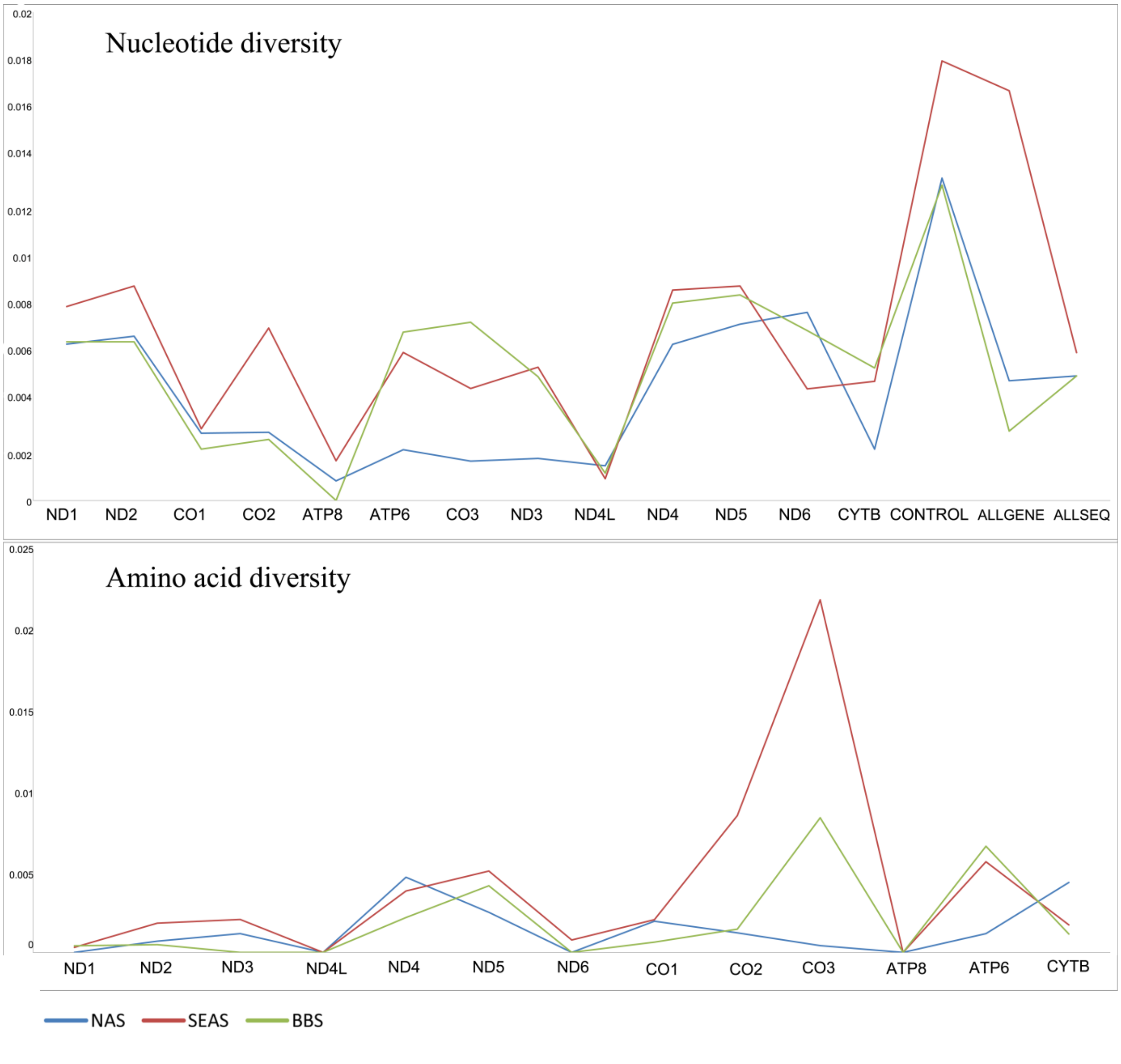

Figure A11. Nucleotide and amino acid diversity of *S. longiceps* populations from 3 ecoregions in the Indian Ocean. NAS (Northern Arabian Sea), SEAS (South Eastern Arabian Sea) and BoB (Bay of Bengal).

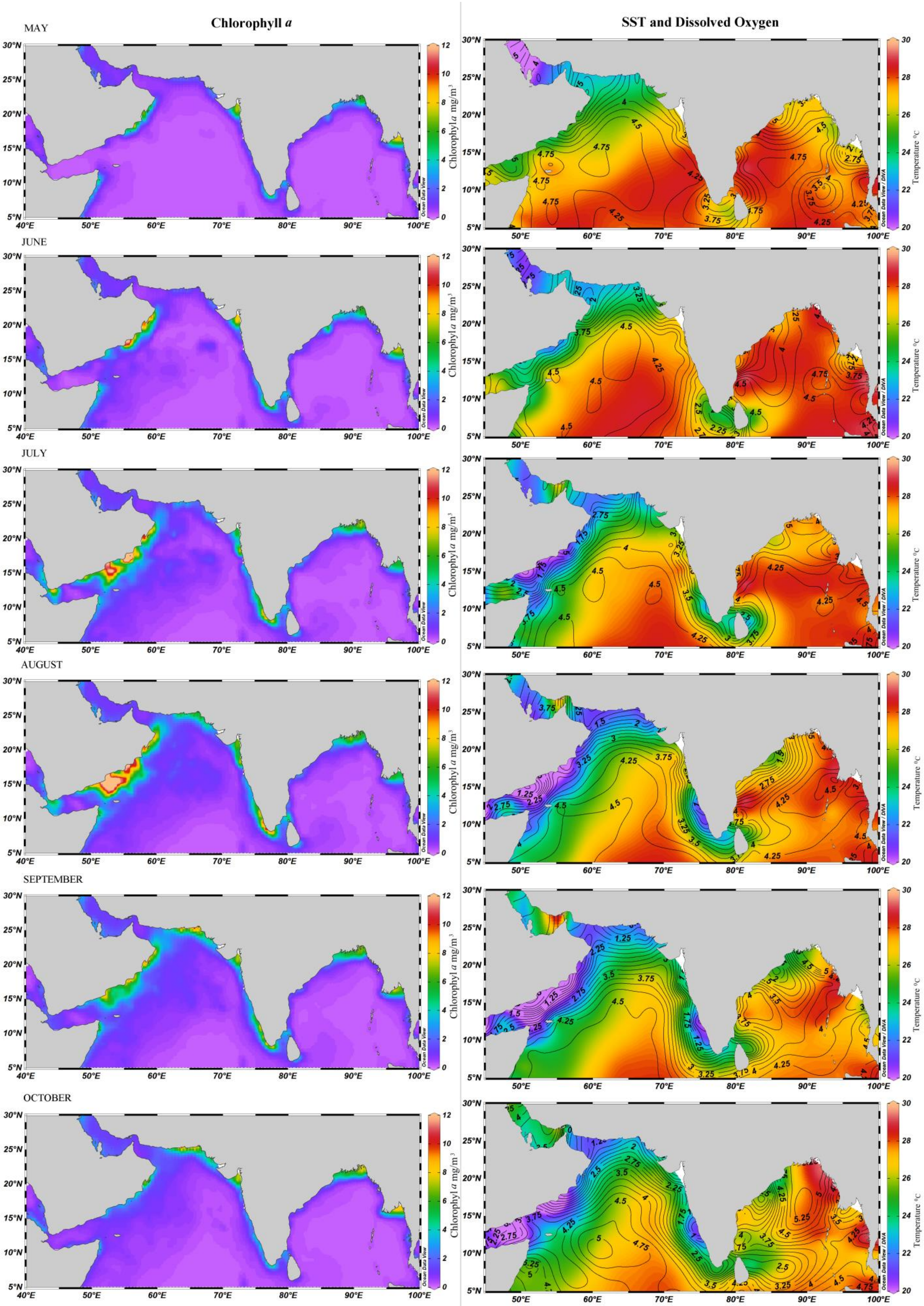

Figure A12. Monthly Chlorophyll *a* (mg/m^3), Sea Surface Temperature- SST (^{0}C) and Dissolved Oxygen (µmol/kg) for the Bay of Bengal and Arabian Ocean during May to October. Chlorophyll *a* and Sea Surface Temperature gradients are represented as coloured shades. Dissolved Oxygen is represented as contour lines.

REFERENCES

Excoffier, L. & Lische, H. E. Arlequin suite ver 3.5, a new series of programs to perform population genetics analyses under Linux and Windows. *Mol. Ecol. Resour*. **10**, 564–567 (2010).
